# Modeling the interactions of sense and antisense *Period* transcripts in the mammalian circadian clock network

**DOI:** 10.1371/journal.pcbi.1005957

**Published:** 2018-02-15

**Authors:** Dorjsuren Battogtokh, Shihoko Kojima, John J. Tyson

**Affiliations:** 1 Department of Biological Sciences, Virginia Polytechnic Institute and State University, Blacksburg, Virginia, United States of America; 2 Biocomplexity Institute, Virginia Polytechnic Institute and State University, Blacksburg, Virginia, United States of America; 3 Division of Systems Biology, Academy of Integrated Science, Virginia Polytechnic Institute and State University, Blacksburg, United States of America; ETH Zurich, SWITZERLAND

## Abstract

In recent years, it has become increasingly apparent that antisense transcription plays an important role in the regulation of gene expression. The circadian clock is no exception: an antisense transcript of the mammalian core-clock gene *PERIOD2 (PER2)*, which we shall refer to as *Per2AS* RNA, oscillates with a circadian period and a nearly 12 h phase shift from the peak expression of *Per2* mRNA. In this paper, we ask whether *Per2AS* plays a regulatory role in the mammalian circadian clock by studying *in silico* the potential effects of interactions between *Per2* and *Per2AS* RNAs on circadian rhythms. Based on the antiphasic expression pattern, we consider two hypotheses about how *Per2* and *Per2AS* mutually interfere with each other's expression. In our *pre-transcriptional* model, the transcription of *Per2AS* RNA from the non-coding strand represses the transcription of *Per2* mRNA from the coding strand and *vice versa*. In our *post-transcriptional* model, *Per2* and *Per2AS* transcripts form a double-stranded RNA duplex, which is rapidly degraded. To study these two possible mechanisms, we have added terms describing our alternative hypotheses to a published mathematical model of the molecular regulatory network of the mammalian circadian clock. Our *pre-transcriptional* model predicts that transcriptional interference between *Per2* and *Per2AS* can generate alternative modes of circadian oscillations, which we characterize in terms of the amplitude and phase of oscillation of core clock genes. In our *post-transcriptional* model, *Per2*/*Per2AS* duplex formation dampens the circadian rhythm. In a model that combines *pre-* and *post-transcriptional* controls, the period, amplitude and phase of circadian proteins exhibit non-monotonic dependencies on the rate of expression of *Per2AS*. All three models provide potential explanations of the observed antiphasic, circadian oscillations of *Per2* and *Per2AS* RNAs. They make discordant predictions that can be tested experimentally in order to distinguish among these alternative hypotheses.

## Author summary

A better understanding of the molecular mechanisms underlying circadian rhythms will undoubtedly improve the treatment of human health problems related to circadian dysrhythmias. However, the inventory of genes and genetic interactions in the circadian clock is still incomplete. Important players may yet be unknown or under-appreciated. For example, in mouse liver, the core clock gene *PER2* is transcribed into both a *Per2* mRNA molecule (a ‘sense’ transcript) and an antisense RNA transcript (*Per2AS*). Because it is important to know how interactions between *Per2* and *Per2AS* may affect circadian gene expression, we have carried out a mathematical modeling study of two possible mechanisms for these interactions. In the *pre-transcriptional* model, *Per2* mRNA interferes with the transcription of *Per2AS* RNA and *vice versa*. In the *post-transcriptional* model, *Per2* and *Per2AS* molecules form double-stranded RNA duplexes, which are rapidly degraded by RNases. We find that the *pre-transcriptional* model gives a more robust account of the circadian, antiphasic oscillations of *Per2* and *Per2AS* transcripts in mouse liver. The model makes an unexpected prediction that co-overexpression of the *ROR* gene and *Per2AS* sequences can generate a new mode of circadian oscillations not seen in contemporary models of circadian rhythms and not yet looked for experimentally.

## Introduction

Messenger RNAs, which encode proteins, are transcribed in the 5'-to-3' direction from one strand (the sense strand) of a structural gene, under the control of an upstream promoter region. For some genes, an ‘antisense’ RNA molecule is transcribed from the opposite strand, driven by an alternative promoter which often lies in an intron of the sense transcript [1, 2]. Antisense transcripts are rarely translated into proteins; their primary effects are in regulating the expression of a ‘target’ transcript [3–6]. Because of their complementary sequences, the natural target of an antisense transcript is typically its sense counterpart and *vice versa*. Interactions between these transcripts are possible not only post-transcriptionally [7–9] but also during the transcription process [10–12]. Difficulties in simultaneously transcribing RNAs from both strands of the same genomic locus, termed transcriptional interference, can mutually repress the expression of both sense and antisense transcripts [13].

Recently Koike *et al*. [14] reported that an antisense transcript of *PER2*, a key core-clock gene, displays oscillatory dynamics. The maximum level of the antisense transcript, *Per2AS*, was about 5% of *Per2*’s maximum level, and the two transcripts were expressed in antiphase, *i*.*e*., the peak of *Per2AS* expression was displaced about 12 h from the peak of *Per2* mRNA. From previous studies of the regulation of gene expression by antisense transcripts in other organisms, it is known that antisense expression can effectively control expression of sense mRNAs; for example, by a tunable, bistable switch [13, 15, 16]. To date the potential regulatory roles of antisense transcripts in a system with oscillatory dynamics have not been studied systematically. Therefore, a natural question is to what extent the rhythms in the mammalian circadian clock can be affected by *Per2AS* expression.

In this work, we study, by numerical simulation and bifurcation analysis [17], the effects of sense-antisense interactions in a mathematical model of the mammalian circadian network proposed by Relogio *et al*. [18]. Relogio’s model is based on two, synergistic feedback loops: the classic, negative feedback loop involving CLOCK/BMAL1 and PER/CRYPTOCHROME (CRY), and the alternative, mixed feedback loop involving BMAL1, REV-ERB (REV) and ROR. We supplement Relogio’s model with an additional, double-negative feedback loop between *Per2* and *Per2AS* RNA species. (Simulations of the original Relogio model agree with many previously reported experimental observations [2, 19–21], and we are careful to retain these successful features of the published model.) By incorporating new terms and variables into Relogio’s model, we study, *in silico*, the effects of two different hypotheses concerning *Per2*-*Per2AS* interactions. In our first model, called the *pre-transcriptional* model, we assume that *Per2* and *Per2AS* mutually repress each other’s production during the process of transcription. This hypothesis is motivated by recent observations of circadian rhythmicity in *Neurospora*, where it was shown that sense and antisense transcripts of the *FREQUENCY (FRQ)* gene control the circadian rhythm by transcriptional interference [22]. Our second model, the *post-transcriptional* model, is based on the assumption that fully transcribed *Per2* and *Per2AS* form double-stranded duplex RNAs, which are degraded by RNases, similar to siRNA- or miRNA-mediated RNA degradation mechanisms [23]. After considering these two models separately, we study a third model that combines pre- and post-transcriptional interactions.

In our simulations of these three modified Relogio-models, the dynamics of *Per2* and *Per2AS* are consistent with the fundamental observation of Koike *et al*. that the RNAs oscillate with ~24 h period and in antiphase to each other. Our *pre-transcriptional* model shows that the interference of *Per2AS* on the transcription of *Per2* and *vice versa* can generate new modes of oscillations (both circadian and non-circadian) in the network, because of the way the double-negative feedback loop between *Per2* and *Per2AS* interacts with the synergistic feedback loops in the original Relogio model. In contrast, the *post*-*transcriptional* model shows that circadian rhythms can be destroyed by *Per2AS* overexpression, because duplex formation rapidly suppresses the expression of *Per2* mRNA. A characteristic feature of the *pre-* and *post-transcriptional* models is that the period of the oscillation is sensitive to the interactions of *Per2* and *Per2AS*. The combined *pre/post-transcriptional* model shows that if *Per2AS* is involved in two different levels of *Per2* regulation, then the period of the oscillation, as a function of *Per2AS* overexpression, can be restricted to a narrow interval.

## Models and methods

### Incorporating sense-antisense transcripts into Relogio’s model of the mammalian circadian clock

**Fig 1:**
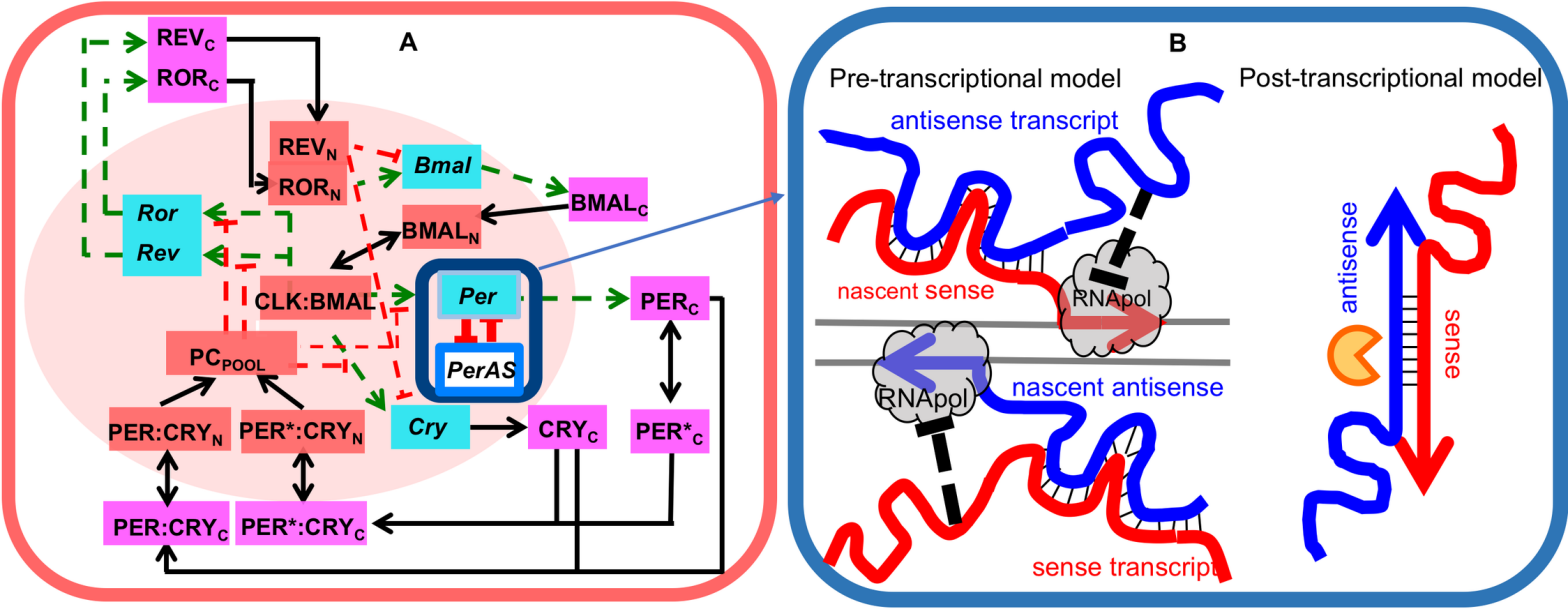
(**A)** Wiring diagram of the circadian clock network in mammalian cells, based on Relogio *et al*. [18]. We have incorporated double-negative interactions between the sense (*Per*) and antisense (*PerAS*) transcripts highlighted by the navy blue outline. Blue boxes mark core-clock genes. Nuclear and cytoplasmic proteins (and protein complexes) are indicated by pink and orange boxes, respectively. The asterisk indicates phosphorylation of PER protein. Black arrows indicate chemical reactions (e.g., phosphorylation and dephosphorylation) or physical translocations (e.g., cytoplasm to nucleus). Dashed lines indicate regulatory effects: activating (green) or inhibiting (red). (**B)** Two hypotheses for sense-antisense RNA interactions. *Pre-transcriptional* hypothesis: *Per2* sense RNA interferes with the transcription of nascent *Per2AS* antisense RNA and *vice versa*. *Post-transcriptional* hypothesis: sense and antisense transcripts form RNA-duplex stretches, where their sequences are complementary. These double-stranded RNA molecules are then rapidly degraded by RNases (the yellow ‘Pac Man’ icon).

Fig 1A presents a schematic diagram of the circadian clock network in mammalian cells, as originally proposed by Relogio *et al*. [18]. CLOCK/BMAL1 up-regulates the expression of the core clock genes, *PER*, *CRY*, *REV*, and *ROR*. Newly synthesized PER and CRY proteins form multimeric complexes in the cytoplasm, and these complexes enter the nucleus, in both phosphorylated and unphosphorylated forms of PER. The PER/CRY complex inhibits CLOCK/BMAL1-activated transcription, by creating a delayed negative-feedback loop in the transcription-translation process. The PER/CRY complex is degraded during the night, releasing its inhibitory effect on CLOCK/BMAL1, to allow a fresh restart of the transcription processes [18].

ROR and REV proteins in the nucleus bind to the promoter region of the *BMAL1* gene, thereby modulating the expression of *Bmal1* mRNA. ROR is an activator and REV an inhibitor of *BMAL1* expression [24]. Previously, *ROR* and *REV* genes were often considered as auxiliary elements in the network, whose primary roles were to fine-tune the expression of *BMAL1* and add robustness to the rhythmic dynamics [25, 26]. However, in the model of Relogio *et al*., the effects of REV and ROR on *BMAL1* expression form independent loops that can generate sustained oscillations autonomously, even if the *PER* and *CRY* genes are expressed constitutively. Some experimental evidence suggests that the feedback loops through *REV* and *ROR* are critical for maintaining circadian oscillations; for instance, when *REV* or *ROR* is overexpressed or both REV-ERBα and REV-ERBβ are knocked-out, circadian rhythmicity can be lost [18, 19, 27].

From the schematic diagram in Fig 1A, Relogio *et al*. derived a system of ordinary differential equations (ODEs) that represent the temporal dynamics of these circadian genes and proteins. Other groups have presented alternative mathematical models of mammalian circadian rhythms [28–31], but the Relogio model is most fitting for our purposes in this paper. In contrast to other models that focus on the negative feedback loop, in which PER/CRY inhibits CLOCK/BMAL1, the Relogio model considers the mammalian circadian clock as a network of synergistic and interlocked feedback loops whereby, in addition to PER/CRY inhibition of CLOCK/BMAL1, REV and ROR control the expression of *BMAL1*, as inhibitor and activator, respectively (see Fig 1A).

The Relogio model [18] consists of 19 ODEs with 76 parameters (rate constants for the constituent biochemical reactions in the network). With an appropriate choice of these parameter values, the model generates simulations in agreement with many well-established experimental properties of circadian rhythms in mammalian cells. For this reason, we have chosen the Relogio model for studying the effects of *Per2* sense-antisense interactions. Our strategy is to incorporate into the model new variables and reaction rates that represent potential interactions of sense-antisense RNAs (*Per2* and *Per2AS*), while keeping the modified model as close as possible to the original Relogio ODEs, and keeping the parameter values as close as possible to the ‘wild-type’ (WT) values in reference [18].

### Hypotheses for sense-antisense transcript interactions

Previously, it was shown by Xue *et al*. in *Neurospora crassa* [22] that coupled transcription of the key circadian gene *FRQ* and its antisense partner *QRF* directly modulates the circadian rhythm, as a consequence of mutually inhibitory interactions between *frq* and *qrf* RNAs. Following this lead, we hypothesize that the interactions of *Per2* and *Per2AS* may also modulate circadian rhythmicity in mammalian cells, by forming a double-negative feedback loop. In Fig 1A, we indicate the mutually inhibitory interactions between *Per* and *PerAS* RNAs by the red lines in a small blue box.

At present, there are no experimental data about the exact molecular mechanisms by which *Per2* and *Per2AS* interact in the circadian network. Therefore, our strategy is to propose reasonable hypotheses for the interaction and to study the consequences of these interactions *in silico*. We propose two simple, feasible mechanisms for sense-antisense interactions, which function either before or after the transcriptional process is complete (Fig 1B). Our aim is not to prove that one or other of these hypotheses is correct, but rather to study the potential effects of sense-antisense interactions on circadian rhythms of the core-clock network, in terms of modulating the period, amplitude, and phases of oscillations.

#### *Pre-transcriptional* model of sense-antisense interactions (Fig 1B, left panel)

First of all, we added a new ODE to Relogio’s model to describe the synthesis and degradation of *Per2AS*:

$$\frac{dPer2AS}{dt}=\frac{\lambda K_{S}}{K_{S}+Per2}-d_{AS}∙Per2AS,$$ (1A)

Eq (1A) includes a simple, phenomenological representation of interference by *Per2* on *Per2AS* transcription: *λ* is the maximum rate of synthesis of *Per2AS* RNA (when antisense transcription is not being interfered with by *Per2* mRNA), and *K*_S_ is the concentration of *Per2* mRNA that causes a 50% decrease in the rate of synthesis of *Per2AS*. Degradation of *Per2AS* is described by the law of mass action, with rate constant *d*_AS_.

In a similar fashion, we modified the ordinary differential equation for *Per2* mRNA dynamics in the original Relogio model (Eq (11) of their Supplemental S1 Text) by multiplying their function, *a∙V*_1max_∙*R*(*…*), for *Per2* transcription by a factor, *μK*_AS_/(*K*_AS_+*Per2AS*), to represent interference by *Per2AS*:

$$\frac{dPer2}{dt}=a∙V_{1max}∙R(\ldots )∙\frac{{\mu K}_{AS}}{K_{AS}+Per2AS}-d_{Per2}∙Per2.$$ (1B)

In this equation, *R*(*…*) is a Hill-type function, 0 ≤ *R*(*k*,*X*) ≤ 1, used by Relogio *et al*. to represent the regulation of *PER2* gene expression by CLOCK/BMAL1 and nuclear PER/CRY (both phosphorylated and unphosphorylated forms):

$$R(\ldots )=\frac{(\frac{1}{a})+{\left(\frac{\left[CLK/BMAL\right]}{K_{t1}}\right)}^{b}}{1+{\left(\frac{\left[CLK/BMAL\right]}{K_{t1}}\right)}^{b}\{1+{\left(\frac{\left[{PER}_{N}/{CRY}_{N}\right]}{K_{i1}}\right)}^{c}\}}$$ (1C)

In Eq (1B) the product *μ*∙*a∙V*_1max_ represents the maximum rate of transcription of *Per2* mRNA. (In Relogio’s model, ‘y1’ is their name of *Per2* mRNA, *d*_y1_ is their name of the rate constant for *Per2* degradation. Their WT values for these parameters are *a* = 12, *V*_1max_ = 1, and *d*_y1_ = 0.3.) For *μ* = 1 and *K*_AS_ >> *Per2AS*, Eq (1B) reduces to the differential equation for *Per2* in the original Relogio model.

We retain the redundancy of parameters (*μ*, *a* and *V*_1max_) that determine the maximum rate of transcription of *Per2* mRNA in order to maintain a certain consistency with the nomenclature and parameter values in the original Relogio model. Relogio *et al*. used *a* to modulate the maximum transcription rate and *V*_1max_ to represent the dosage of the *PER2* gene (hence, *V*_1max_ = 1 in the WT parameter set). We retain that distinction, and we introduce a third parameter, *μ*, to represent the strength of the interference of *Per2AS* on the transcription of *Per2* mRNA. Nonetheless, one should remember that it is always the product *μ*∙*a∙V*_1max_ that determines the maximum rate of transcription of *Per2* mRNA in the differential equations.

We could consider Eqs (1A) and (1B) as simple, phenomenological representations of the mutual interference between *Per2* and *Per2AS* at the level of gene transcription, without being specific about the precise mechanism of these interactions. However, as we show in Suppl. S1 Text, we can derive Eqs (1A) and (1B) from a detailed molecular model (Suppl. S1 Fig) of transcriptional interference, as suggested by the left panel of Fig 1B. In either interpretation, Eqs (1A) and (1B) are suitable mathematical representations of the effects of transcriptional interference on the dynamics of the circadian rhythm model in mammalian cells.

We note that our *pre-transcriptional* model differs from a model proposed previously by Xue *et al*. [22] for interactions between the sense (*FRQ*) and anti-sense (*QRF*) transcripts of a circadian rhythm in *Neurospora*. Although these authors attribute the interactions to “premature termination of transcription”, they model the interactions (in their Extended Data Fig 4C) as a loss of *FRQ* RNA at a rate proportional to *QRF* concentration and *vice versa*. In our formulation, the rate of synthesis of *FRQ* RNA decreases with increasing *QRF* concentration (and *vice versa*) but never becomes negative. Our formulation of the rate equations is more realistic biochemically, and it produces results that are consistent with the reported dynamics of *frq* and *qrf* genes in the previous work.

#### *Post-transcriptional* model of sense-antisense interactions (Fig 1B, right panel)

In this version of the model, we assume that, due to their complementary sequences, sense-antisense transcripts form duplexes, *i*.*e*., double-stranded RNAs. Because duplex formation reduces the levels of both transcripts, it can be considered as a mutually inhibitory interaction. Letting *k*_assn_ be the rate constant for duplex formation (association) and *k*_diss_ the rate constant for the reverse reaction (dissociation), we write our model for *post*-*transcriptional* interactions as:

$$\begin{array}{l} \;\frac{dPer2AS}{dt}=\lambda_{0}-k_{assn}∙Per2AS∙Per2+k_{diss}∙Dplx-d_{AS}∙Per2AS,\\ \;\frac{dDplx}{dt}=k_{assn}∙Per2AS∙Per2-k_{diss}∙Dplx-d_{dup}∙Dplx,\\ \frac{dPer2}{dt}={a∙V}_{1max}∙R(\ldots ){-k_{assn}∙Per2AS∙Per2+k_{diss}∙Dplx-d}_{Per2}∙Per2. \end{array}$$ (2)

In this version of the model, *λ*_0_ is the (constant) synthesis rate of *Per2AS* and *d*_dup_ is the rate constant for degradation of the duplex RNA. In this formulation of the model, degradation of the duplex RNA provides an alternative pathway for loss of *Per2* and *Per2AS* RNAs, without introducing the possibility of negative RNA concentrations, as in the differential equations proposed by Xue *et al*. [22].

#### Combined *pre/post* model for irreversible duplex formation

We also consider a model that combines *pre-* and *post*-*transcriptional* interactions. For simplicity, we assume in the combined model that *d*_dup_ >> 1 or *k*_diss_ << 1, so that the formation of duplex sense-antisense molecules can be considered an irreversible loss of single-stranded RNAs. This case is described by the following two ODEs,

$$\begin{array}{l} \;\frac{dPer2AS}{dt}=\lambda_{0}+\frac{\lambda_{1}K_{S}}{K_{S}+Per2}-k_{assn}∙Per2AS∙Per2-d_{AS}∙Per2AS,\\ \frac{dPer2}{dt}={a∙V}_{1max}∙R(\ldots )∙\frac{\mu K_{AS}}{K_{AS}+Per2AS}-k_{assn}∙Per2AS∙Per2-d_{Per2}∙Per2. \end{array}$$ (3)

### Numerical methods

Numerical simulations were carried out in Mathematica, and bifurcation diagrams were calculated using AUTO [17]. In some circumstances, parameter values in the models were fitted to experimental data using the ensemble method [32] described in Suppl. S4 Text.

## Results

### Analysis and simulation of the *pre-transcriptional* model

The differential equations of the *pre-transcriptional* model (*i*.*e*., Relogio’s differential equations supplemented with Eqs (1A) and (1B)) are provided in Suppl. S2 Text. The parameter values proposed by Relogio et al. [18] are listed in Suppl. S1 Table, where they are called ‘WT’ values. Suppl. S1 Table also lists proposed values for the parameters *μ*, *λ*, *K*_S_ and *K*_AS_ that characterize the mutual interference between *Per2* and *Per2AS*.

#### Simulations of Koike *et al*. observations

**Fig 2:**
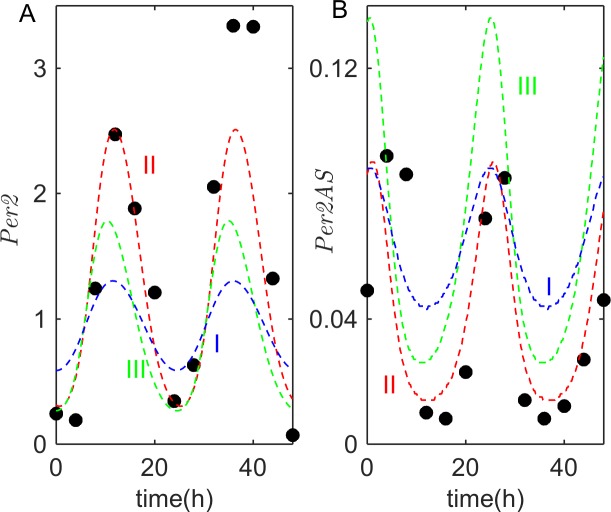
Simulations of *Per2* and *Per2AS* oscillations in the *pre-transcriptional* model. Solid circles mark time-courses of *Per2-*sense (left) and -antisense (right) transcripts, as observed by Koike *et al*. in mouse liver [14]. Blue, green, and red lines show model simulations for three alternative sets of parameter values provided in Suppl. S1 Table. Among these parameter sets, Set III simulations are closest to simulations of the model with Relogio’s WT parameter values (see Suppl. S2 Fig).

Koike *et al*. [14] recently reported a large volume of RNA-seq data which show rhythmic dynamics of many genes, including known core clock genes. These data will be very useful for building large, more comprehensive models of circadian rhythms in mammals. In this work, however, our aim is limited to explaining their observations of antiphasic oscillations of *Per2* and its antisense transcript, *Per2AS*. Using the WT parameter values in Suppl. S1 Table, we find that our model does indeed provide a good fit to Koike’s data (see Fig 2) and is also consistent with the observed phases of maximal expression of core-clock genes (see Fig 4C for *λ =* 1), as catalogued in Relogio *et al*. [18].

In Fig 2, we show *Per2* and *Per2AS* time-courses for three other sets of parameter values (see Suppl. S1 Table), to demonstrate the robustness of the *pre-transcriptional* model. In these simulations, we allowed all of the parameters in the model to vary, and we fitted the simulations to the observations of Koike *et al*. [14] and to the data reported in Relogio *et al*. [18] for both knock-out and over-expression mutants, as well as the expression phases of the core clock genes using an ensemble method [32]; see Suppl. S4 Text. From these results, we conclude that our *pre-transcriptional* model provides a robust description of the known properties of circadian gene expression in murine cells with minimal modifications to the original Relogio model.

#### Bifurcation analysis

**Fig 3:**
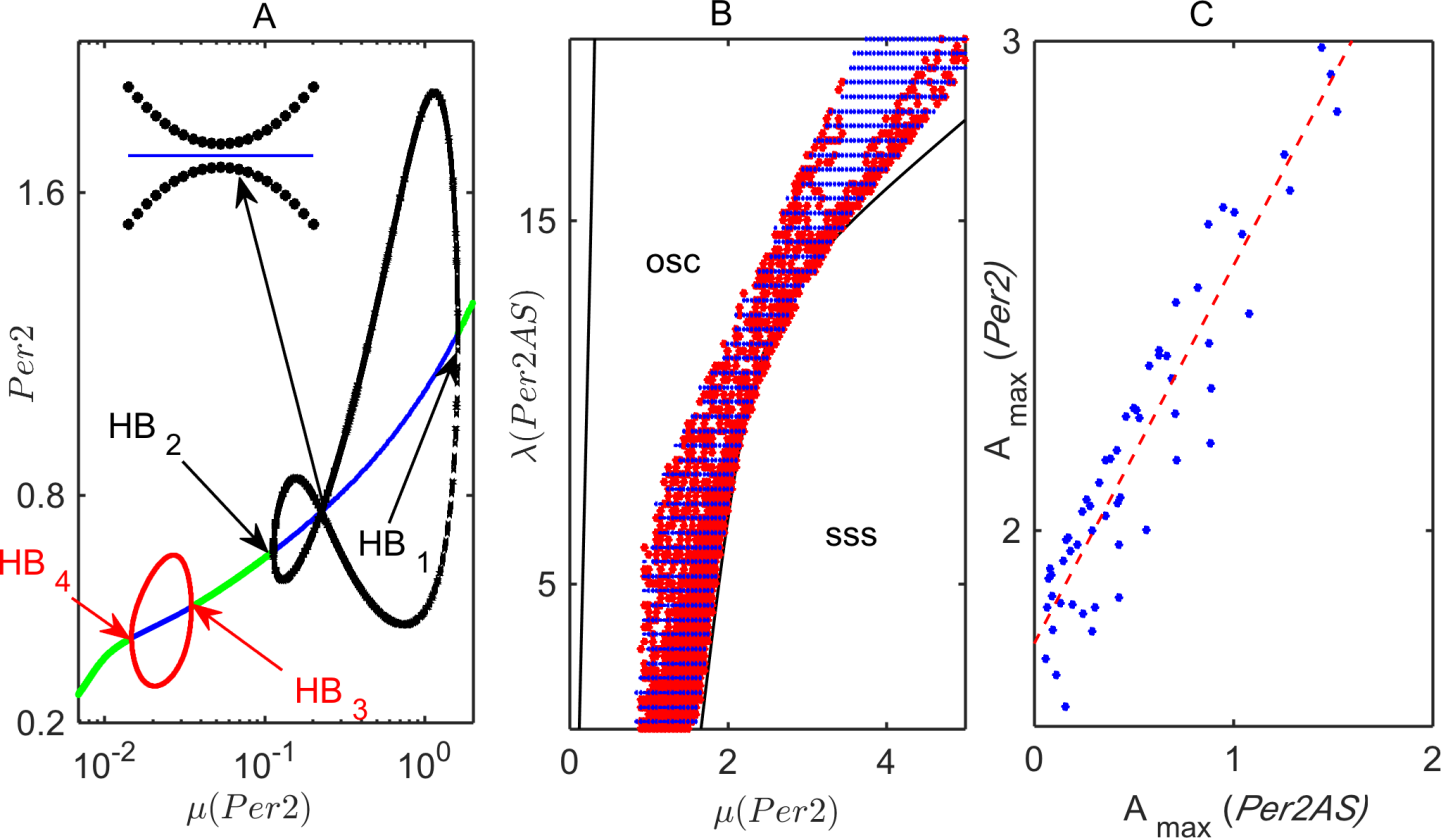
Bifurcation analysis of the *pre-transcriptional* model. **(A)** One-parameter bifurcation diagram. The transcription rate of the *Per2* mRNA is varied, using *μ* as the primary bifurcation parameter, while all other parameters are fixed at Relogio’s WT values (Suppl. S1 Table); in particular, *λ =* 1. Green lines indicate stable steady states; blue lines, unstable steady states. In this diagram there are four Hopf bifurcation points, HB_*i*_, *i* = 1,…,4. The black and red curves indicate the amplitude (maximum and minimum) of the oscillations that bifurcate from the HB points. (**B**) Two-parameter bifurcation diagram on the (*μ*, *λ*) parameter plane. Solid black lines are continuations of the Hopf bifurcation points HB_1_ and HB_2_ in panel A. Blue symbols represent parameter combinations that give a circadian rhythm, 23.2 h < *T* < 23.7 h. Red symbols mark the region where, in addition to this restriction on oscillation period, *Per2* and *Per2AS* oscillate nearly out of phase, *i*.*e*., 11 h < |*ϕ*_*Per2*_ –*ϕ*_Per2AS_| < 13 h. Other parameters are fixed at the WT ‘Relogio’ values. (**C**) The relationship between the maximum amplitudes (*A*_*max*_) of *Per2* and *Per2AS* oscillations for a sample of parameter combinations shown in panel B by the red symbols. The dashed red line is a linear regression to the sample points.

In this subsection, we use bifurcation theory [17, 33] to gain a better understanding of the effects of *Per2* and *Per2AS* interactions in the *pre-transcriptional* version of the Relogio model. Fig 3A shows a one-parameter bifurcation diagram, using *μ* as the primary bifurcation parameter. All other parameters are fixed at their WT values in the original Relogio *et al*. publication; see Suppl. S1 Table. (Recall that the maximum rate of *Per2* transcription is *a*∙*V*_1max_∙*μ* = 12*μ* for WT values of *a* and *V*_1max_.) There are four Hopf bifurcation points, HB_1_ to HB_4_, in Fig 3A. The oscillations between HB_3_ and HB_4_ are slow (period ≈ 50 h), whereas the oscillations between HB_1_ and HB_2_ are circadian (period ≈ 23.5 h). The amplitude of these oscillations reaches its maximum at *μ* ≈ 1.1. An interesting feature of the bifurcation diagram in Fig 3A is that, at *μ* ≈ 0.12 (see inset in Fig 3A), the amplitude of *Per2* oscillations (max–min) almost vanishes, due to multiple, competing, repressive feedbacks exerted on *Per2*.

In Fig 3B, we plot a two-parameter bifurcation diagram on the (*μ*,*λ*) parameter plane. The *pre-transcriptional* model exhibits antiphasic, circadian oscillations over a broad range of the rate constant, *λ*, for the synthesis of *Per2AS* RNA, despite the assumption that *Per2AS* transcription represses the production of *Per2* mRNA. Furthermore, when *Per2* and *Per2AS* oscillations are circadian and antiphasic, their amplitudes are positively correlated (Fig 3C), despite their mutually repressive interactions.

**Fig 4:**
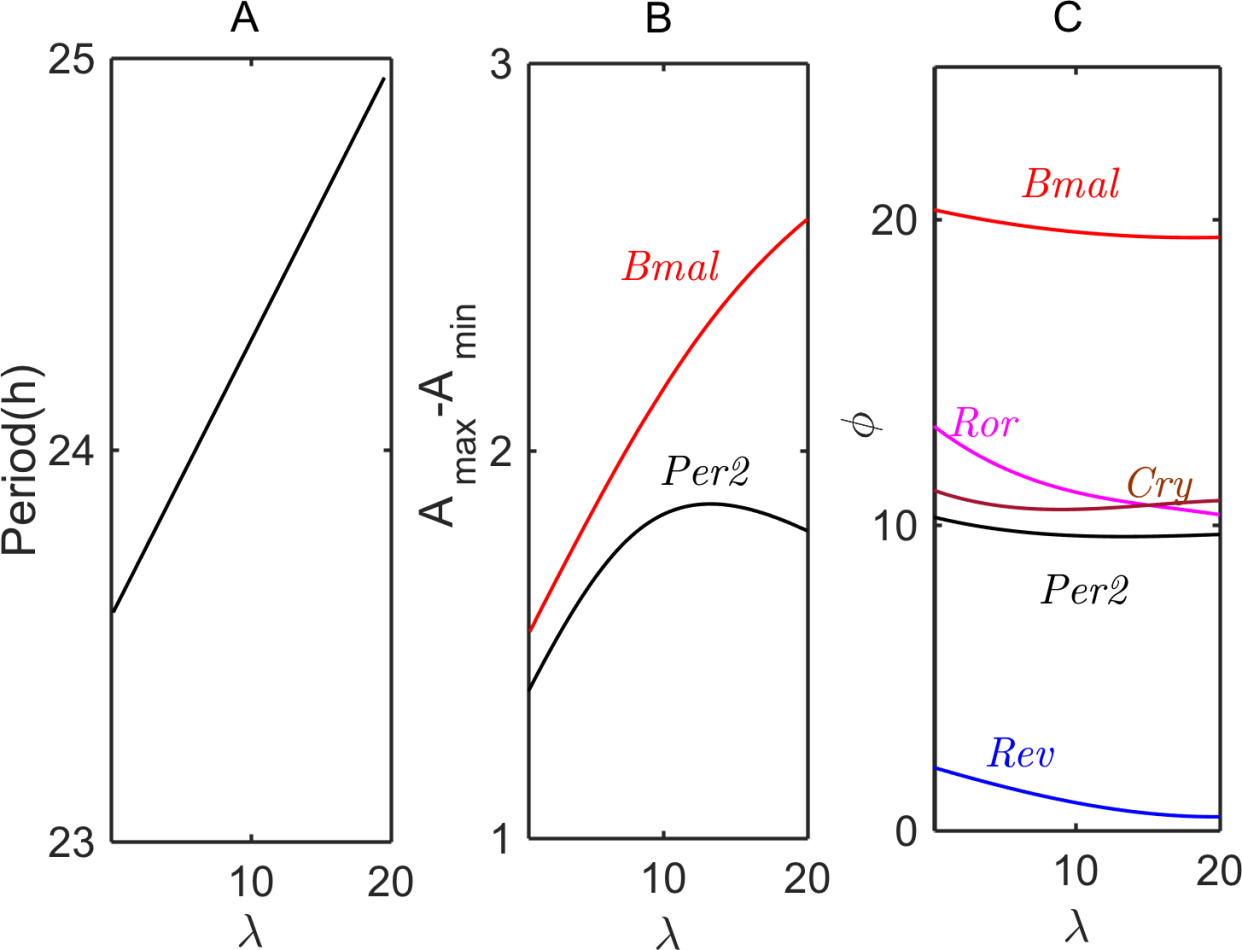
**Modulations of period (A), amplitude *A***_***max***_***−A***_***min***_ **(B), and phases *ϕ* (C) of oscillation with increasing value of *λ*, the synthesis rate of *Per2AS*.** In panel C, the phase of *Per2AS* is fixed at 0.

Fig 4 shows how, in the *pre-transcriptional* model, the period and amplitude of *Per2* and *Bmal1* oscillations, as well as the phases of oscillation of the core-clock genes, change with increasing value of *λ*, the maximum synthesis rate of *Per2AS* (see Eq (1A)). The period of oscillation (Fig 4A) increases linearly with *λ*. The amplitude of *Per2* oscillations initially increases with increasing *λ* (Fig 4B), because the inhibition of *Per2* by *Per2AS* releases the repression of the transcriptional activator (CLOCK/BMAL1) by the PER/CRY complex. Hence, as the activity of CLOCK/BMAL1 increases, the levels of other core clock genes also increase. However, as Fig 4B shows, at sufficiently large values of *λ*, the increase of *Bmal1* level slows, and *Per2* level starts to drop. Meanwhile, the phases of maximum expression of core clock genes change only slightly with increasing *λ* (Fig 4C). The most notable phase changes are evidenced by *Ror* and *Rev*.

#### Emergent oscillations

**Fig 5:**
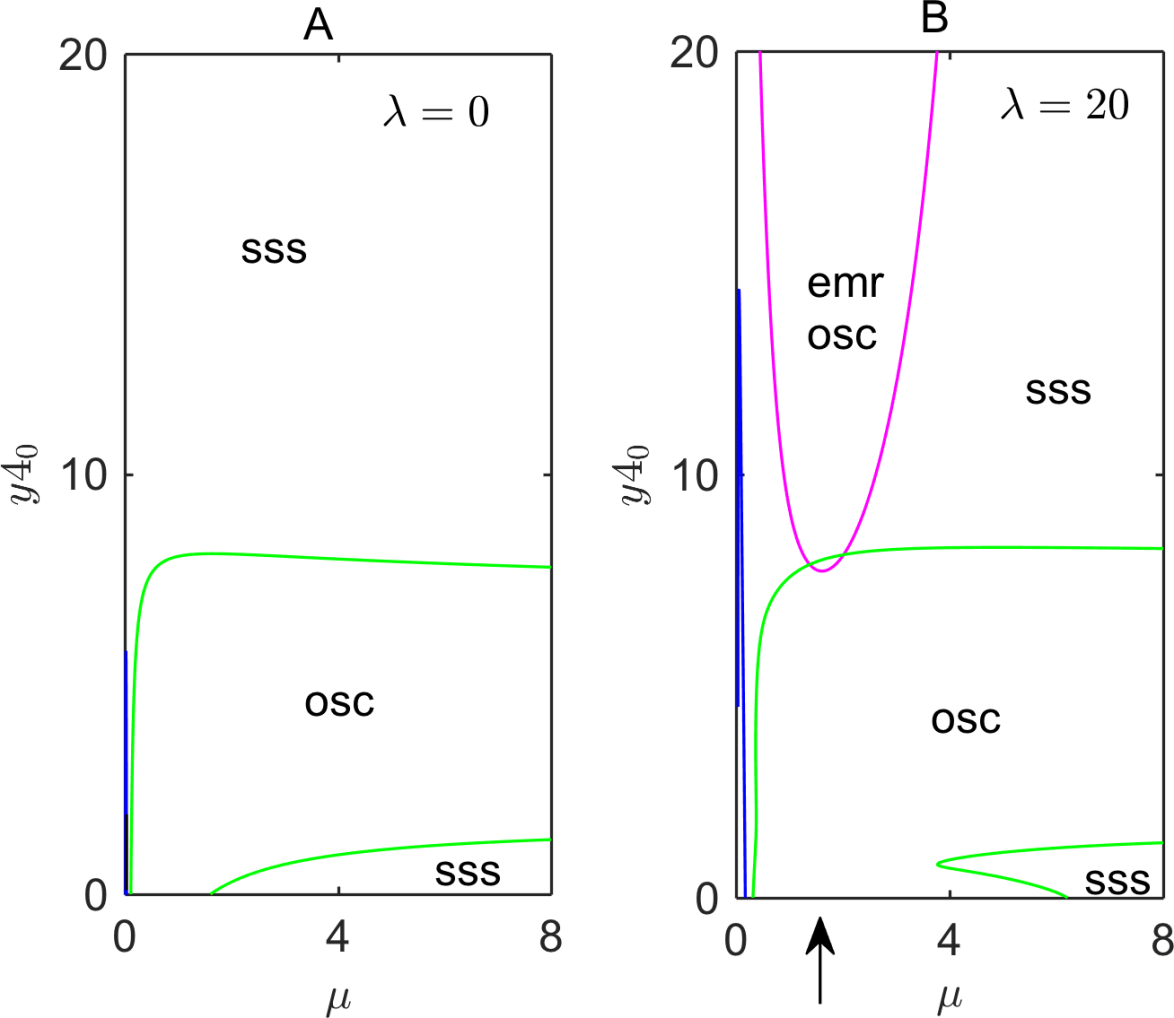
Two-parameter bifurcation diagrams with exogenously overexpressed *Ror*. The two parameters are *μ* (proportional to the maximum rate of synthesis of endogenous *Per2*) and *y4*_0_ (the constant rate of synthesis of *Ror* from a plasmid). (**A)** *Per2AS* is absent (*λ* = 0); or (**B)** *Per2AS* is overexpressed (*λ* = 20). The green and blue lines are continuations of the Hopf bifurcation points marked by HB_1_ and HB_2_ and by HB_3_ and HB_4_, respectively, in Fig 3A. The purple line in panel B shows the domain of emergent oscillations (emr osc) at *λ* = 20. The up-arrow indicates the value of *μ* chosen for the calculations in Fig 6.

We use two-parameter bifurcations diagrams (Fig 5) to explore the effects of *Per2AS* transcription on other elements of the core clock network, in the *pre-transcriptional* model. Fig 5A shows a two-parameter bifurcation diagram for the original Relogio model in the parameter plane spanned by *μ* (the maximal rate of synthesis of *Per2* mRNA) and *y4*_0_ (the rate of transcription of *Ror* mRNA from an exogenous *ROR* gene, e.g., carried by a plasmid). A prediction of the Relogio model is that circadian oscillations should disappear for *y4*_0_ greater than ~8; a prediction that was confirmed by constitutive overexpression of *Ror* from an exogenous copy of the gene [18]. In contrast to this prediction, we find, in the *pre-transcriptional* model, a new region of ‘emergent’ rhythmic dynamics (bounded by the purple curve in Fig 5B), attributable to the double-negative feedback loop between *Per2* and *Per2AS*, when the production rate of *Per2AS* is large enough (*λ* = 20, in Fig 5B). In contrast to exogenous overproduction of *Ror* alone, which leads to damped oscillations of *Bmal1*, our model predicts that double overproduction of *Ror* and *Per2AS* restores stable circadian oscillations.

**Fig 6:**
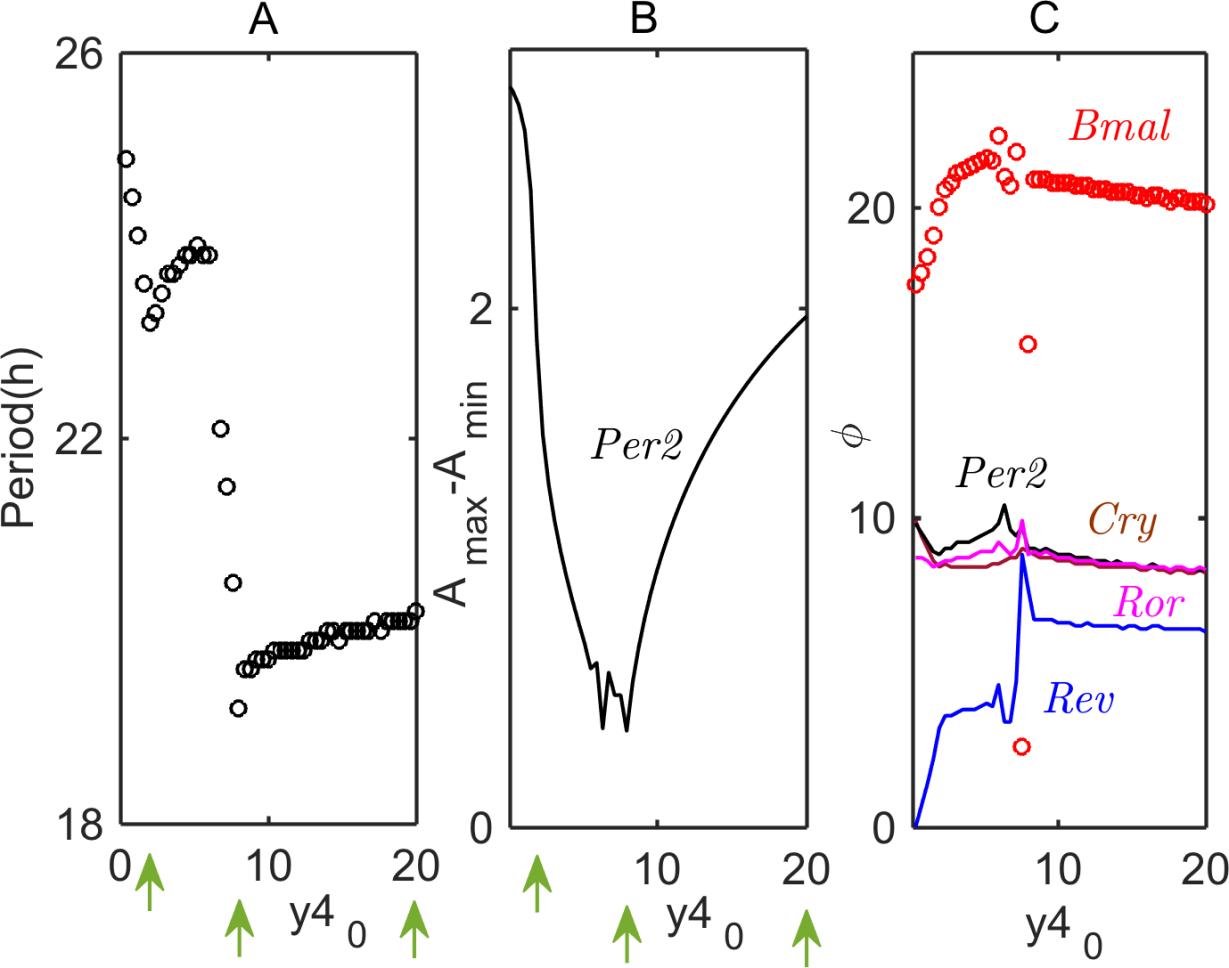
**Modulations of the period (A), amplitude (B), and phases (C) of oscillation with an increase of the constitutive rate of synthesis of *Ror* from a plasmid (*y4***_**0**_**), for a fixed rate of overexpression of endogenous *Per2AS* (*λ =* 20).** In these simulations, *μ* = 1.6. The up-arrows indicate the values of *y4*_0_ for which oscillations are plotted in Suppl. S3 Fig.

In Fig 6 we simulate changes of the period, amplitude, and phases of oscillation with an increasing value of *y4*_0_, when other control parameters are fixed, in particular, *λ* = 20 and *μ* = 1.6 (refer to the up-arrow in Fig 5B). The period of oscillation drops sharply at *y4*_0_ ≈ 7–8, where the system approaches the region marked by the upper green line in Fig 5. In the original Relogio model, at this value of *y4*_0_, the oscillatory dynamics vanishes. However, if *Per2AS* is overexpressed, the system transitions to a different oscillatory domain engendered by *Per2*-*Per2AS* interactions. Fig 6A shows that the period of the emergent oscillations is about 20 h. Fig 6B shows that the maximum amplitude of *Per2* oscillations changes drastically with an increase of *y4*_0_. Discontinuous jumps of the oscillation phases (Fig 6C) confirm the existence of two independent oscillatory regimes in the system.

Fig 6 shows that, in the *pre-transcriptional* model, with increasing values of the parameter *y4*_0_ controlling exogenous expression of *Ror*, the system transitions from one domain of oscillations to another, if *Per2AS* is also overexpressed (*λ* = 20). To elaborate on this effect, we show, in Suppl. S3 Fig, temporal patterns of *Per2* and *Per2AS* at three different values of *y4*_0_ (see the up-arrows in Fig 6) over a time interval of 200 h. When *y4*_0_ is small (*y4*_0_ = 1), the period of the oscillations is about 25 h, and the amplitude of *Per2* oscillations is large (Suppl. S3A and S3B Fig). Because *λ* is large, the amplitude of *Per2AS* oscillations is also large. However, at *y4*_0_ = 8, the amplitude of *Per2* and *Per2AS* oscillations drop significantly, and the temporal patterns are bimodal (Suppl. S3C and S3D Fig), because near this value of *y4*_0_, the system is at the interface of two different oscillatory domains. When the system is deep inside the second domain (at *y4*_0_ = 20), the period of oscillations is ~21 h, and the amplitudes are large again (Suppl. S3E and S3F Fig).

In Suppl. S4 Fig, we show that circadian oscillations can also be abolished by constant high level of REV-ERB in the nucleus (*x5*(*t*) = *x5*^0^ = constant), and then restored by overexpression of endogenous *Per2AS* (the parameter *λ*). In Suppl. S5 Fig, we compare the distributions of phases of clock-gene expression in the mutant compared to a WT cell. In Suppl. S6 Fig, we show the domains of oscillations in a two-parameter bifurcation diagram, using *x5*^0^ and *λ*, as bifurcation parameters. The open red circle in Suppl. S6 Fig shows the case of a stable steady state at *x5*^0^ = 2.4 and *λ* = 0 (Suppl. S4 Fig). As first reported in Ref. [18], when *x5*^0^ is increased further, oscillations reappear in the Relogio model, but their period is considerably longer than 24 h. A new, emergent domain of oscillations is found only when *Per2AS* is strongly expressed. For certain values of the parameters in the model, the period of the oscillation is ~24 h (see Suppl. S4 Fig).

### Analysis and simulation of the *post-transcriptional* model

**Fig 7:**
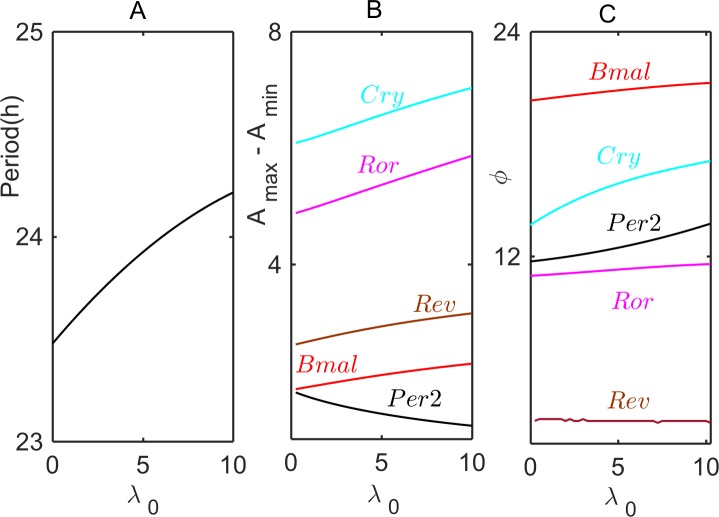
**Modulations of period (A), amplitudes (B), and phases (C) of oscillation of core clock genes with increasing value of the rate of exogenous synthesis of *Per2AS* in the *post-transcriptional* model.** *Per2AS* overexpression dampens the amplitude of *Per2* oscillations; consequently, because of the release of PER/CRY repression of CLOCK/BMAL1, the levels of other core clock genes increase. Parameter values are *k*_assn_ = 0.1, *k*_diss_ = 0.1, *d*_dup_ = 0.1; other parameters in the Relogio model retain their WT values. The phase of *Per2AS* in panel C is fixed at 0.

In the *post-transcriptional* model (the Relogio model modified by Eq (2)), we assume that the physical interaction (duplex formation) between sense-antisense transcripts causes mutual degradation of both RNAs. In this case, the amount of *Per2AS* in a cell is especially important, and this amount is determined by the parameter *λ*_0_, which represents constitutive transcription of *Per2AS* from both the endogenous *PER2AS* sequence and from exogenous *Per2AS* sequences carried on a plasmid. In Fig 7 we show how the period, amplitudes and phases of the rhythm depend on the value of *λ*_0_. In these simulations, unless otherwise specified, all parameters of the Relogio model are fixed at their WT values, and the additional parameters in Eq (2) are fixed at *k*_assn_ = 0.1, *k*_diss_ = 0.1, and *d*_dup_ = 0.1.

We assume that the contribution to *λ*_0_ from the endogenous gene is small (say, 0.1 < *λ*_0_ < 1) compared to the contribution due to plasmid copies of *Per2AS* sequences (say, *λ*_0_ > 1). At *λ*_0_ = 0.2 (representative of endogenous synthesis only), the period of the oscillations in the *post-transcriptional* model is ~23.5 h, the maximum level of *Per2AS* is about 5% of the maximum level of *Per2*, and *Per2* and *Per2AS* oscillate out-of-phase, *i*.*e*. |ϕ_*Per2*_ − ϕ_*Per2AS*_| ≈ 12 h (see Suppl. S7 Fig). In other words, at these parameter values, the *post-transcriptional* model exhibits oscillations that fit reasonably well the time-courses of *Per2* and *Per2AS* oscillations observed by Koike *et al*., shown by the black circles in Fig 2.

Fig 7 shows how the properties of circadian rhythms change in the *post-transcriptional* model with increasing rates of synthesis of exogenous *Per2AS* (parameter *λ*_0_). The period of oscillations increases modestly with increasing *λ*_0_ (Fig 7A). The amplitude of *Per2* oscillations drops with increasing *λ*_0_, because of the duplex formation, whereas the amplitudes of oscillation of other core-clock genes increase (presumably CLOCK/BMAL1 is less strongly repressed by PER/CRY) (Fig 7B). With *Per2AS* phase set at 0 hours, we plot in Fig 7C the changes in the phases of oscillation of core clock genes. Blue and black lines in Fig 7C show that the phases of *Per2* and *Cry* mRNAs are most sensitive to the increase of *Per2AS* level.

In Suppl. S8A Fig we plot a two-parameter bifurcation diagram on the parameter plane (*λ*_0_, *k*_assn_). Although the oscillatory domain is very large in this diagram, the region where the *post*-*transcriptional* model oscillates with circadian properties is restricted; the black symbols mark the region where following conditions are fulfilled:

$$\begin{array}{l} 23\;h<T<25\;h,\\ (A_{max}^{Per2}-A_{min}^{Per2})>0.5,\\ {11\;h<|\phi }_{Per2}-\phi_{Per2AS}|<13\;h. \end{array}$$ (4)

**Fig 8:**
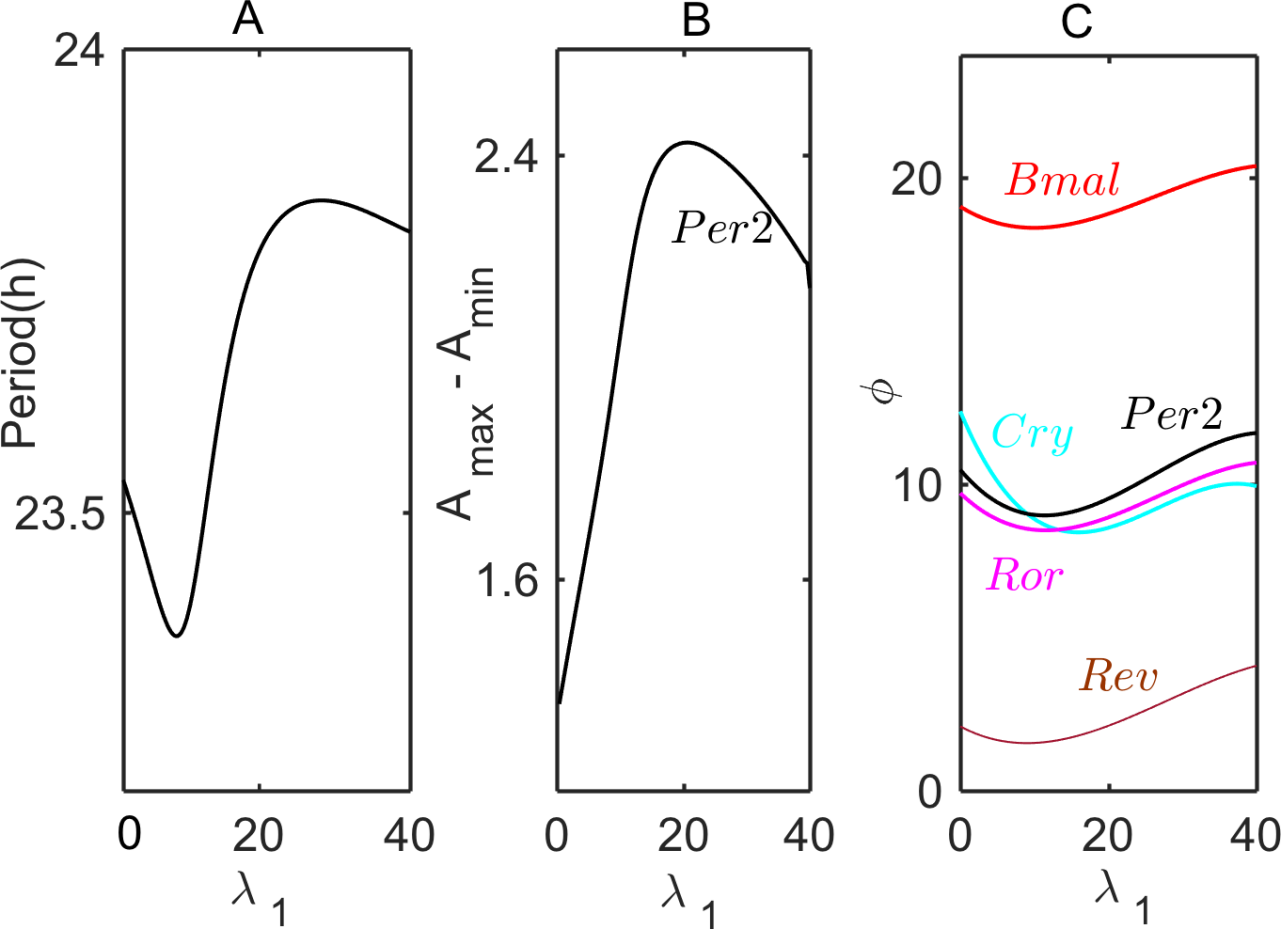
**Modulations of the period (A), amplitude (B), and phases (C) of oscillations in the combined *pre/post-transcriptional* model.** Parameter values are: *λ*_0_ *=* 0, *k*_assn_ *=* 0.1, *μ* = 1; all others are fixed at WT values. In panel C, the phase of *Per2AS* is fixed at 0.

In Suppl. S8B Fig we plot a two-parameter bifurcation diagram on the parameter plane (*d*_dup_, *k*_assn_), while fixing *λ*_0_ = 10 and *k*_diss_ = 0.1. The non-oscillatory domain in the middle of the diagram separates a region of circadian oscillations (22 h < *T* < 25 h) at the bottom of the diagram from a region of slow oscillations (*T* > 50 h) at the top. The region of this diagram where conditions in Eq (4) are fulfilled (marked by small red symbols) is quite restricted: 0.08 < *d*_dup_ < 0.27 and 0 < *k*_assn_ < 0.2. The blue symbols in Suppl. S8B Fig mark the region where the first and second conditions of Eq (4) are fulfilled, but the oscillations of *Per2* and *Per2AS* are not strictly antiphasic, *i*.*e*., 9 h < |ϕ_Per2_ − ϕ_Per2AS_| < 15 h.

In Suppl. S9 Fig, we plot the time-courses of oscillations at three locations in Suppl. S8B Fig. Suppl. S9A–S9C Fig show the case: *k*_assn_ = 1, *d*_dup_ = 0.1 for two values of *λ*_0_. When *λ*_0_ = 1, the dynamics of *Per2* is reminiscent of WT dynamics in the Relogio model, but when *λ*_0_ = 10, the amplitude of *Per2* oscillations has become very small. For the case *k*_assn_ = 1, *d*_dup_ = 0.2 (Suppl. S9D–S9F Fig), the amplitudes of oscillations at *λ*_0_ = 10 are larger, but the waveform has become distinctly non-harmonic. For the case *k*_assn_ = 5, *d*_dup_ = 0.2 (Suppl. S9G–S9I Fig), the amplitudes of oscillations at *λ*_0_ = 10 are quite large, the waveforms are very non-haromonic, and the period (~30 h) is non-circadian.

Our explorations of the *post-transcriptional* model show that it can be parameterized to fit the observations in Koike *et al*. [14], but the range of suitable parameter values is restricted. If any of the parameters *d*_dup_, *k*_assn_, or *k*_diss_ in Eq (2) deviate too much from the preferred values, the oscillations may no longer fulfill the requirements in Eq (4). Especially if *k*_diss_ > *k*_assn_, the peak amplitudes of *Per2* and *Per2AS* become quickly non-antiphasic. Therefore, we conclude that the formation of *Per2*/*Per2AS* duplex RNA tends to destroy circadian rhythms over a wide range of values of the parameters *d*_dup_, *k*_assn_, and *k*_diss_.

### Analysis and simulation of a combined *pre/post-transcriptional* model

Eq (3) details how we modified the Relogio model to include both *pre-* and *post-transcriptional* interactions of *Per2* and *Per2AS*. Fig 8 shows how the period, amplitude, and phases of circadian oscillations change with increasing *λ*_1_ for fixed *λ*_0_ = 0, *μ* = 1 and *k*_assn_ = 0.1. The combined model is consistent with circadian, antiphasic oscillations of *Per2* and *Per2AS* (see Suppl. S10 Fig). Unlike simulations of the *pre-* or *post-transcriptional* model, shown in Figs 4 and 7, the period, amplitude and phases of oscillation in the combined model are distinctly non-monotonic in dependence on *λ*_1_.

**Fig 9:**
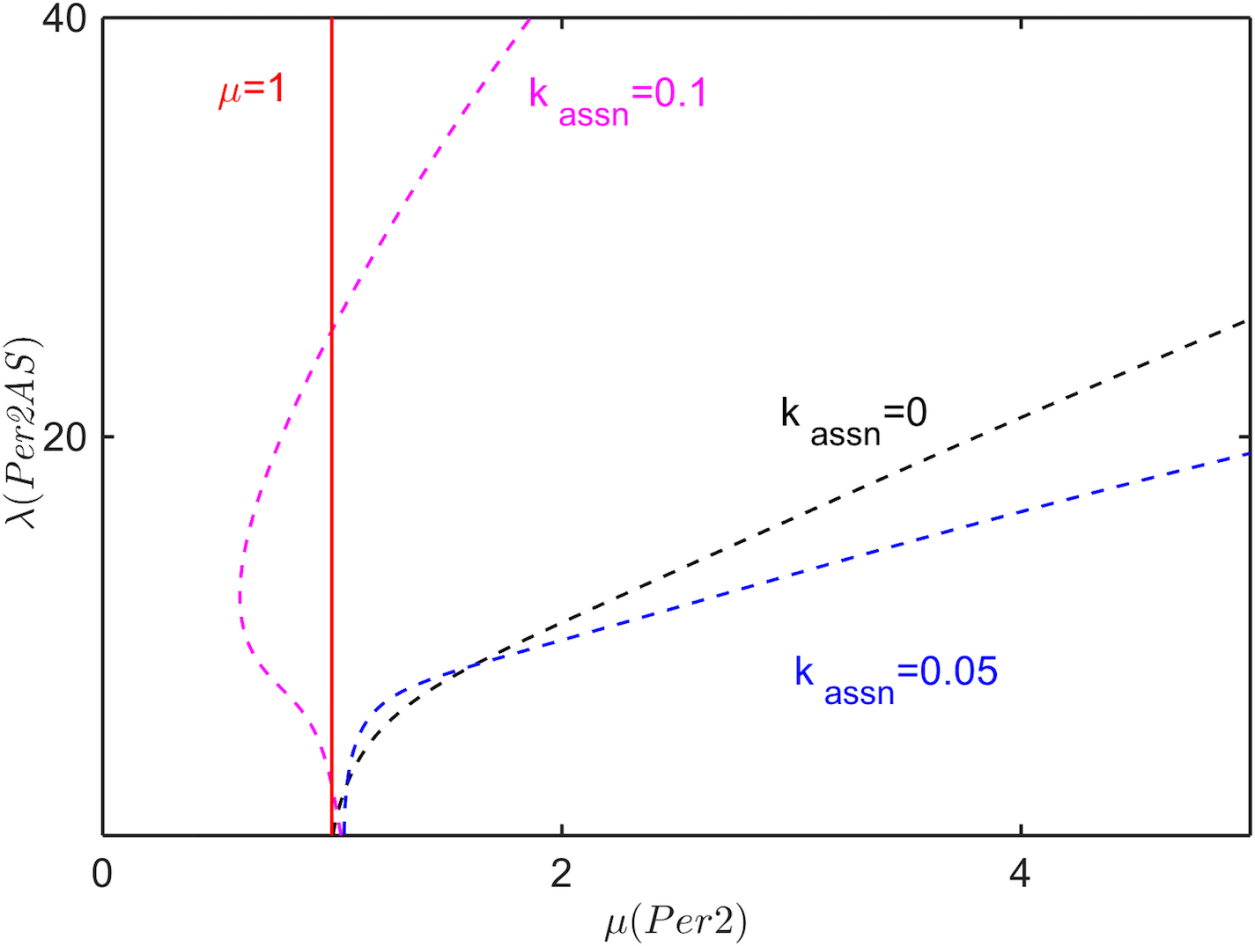
Two-parameter bifurcation diagram of a combined *pre/post-transcriptional* model. Dashed lines show the continuations of limit cycle oscillations of period *T* = 23.5 h at three different values of the duplex-formation rate constant: *k*_assn_ = 0 (black), 0.05 (blue) and 0.1 (purple). In these calculations, *λ*_0_ = 0 and other parameters are fixed at their WT values.

On Fig 9 we continue the limit cycle oscillations of period *T* = 23.5 h on the parameter plane (*μ*, *λ*) for three different values of the rate constant for duplex formation, *k*_assn_. Notice that, compared to the case *k*_assn_ = 0 (*i*.*e*., no duplex formation), the locus of 23.5-hour rhythms does not change much for *k*_assn_ = 0.05, but it is radically different for *k*_assn_ = 0.1, intersecting the line *μ* = 1 twice, at *λ* ≈ 1 and *λ* ≈ 25. Therefore, as Figs 8 and 9 show, the combined *pre/post-transcriptional* model can restrict the period of oscillations within tighter bounds of *μ*. The reason is that, unlike the *pre-* or *post-transcriptional* model for which *Per2*-*Per2AS* interactions directly modulate only a single process of gene regulation, in the combined model two different gene-regulatory processes are simultaneously modulated. As a result, due presumably to counter-balancing effects, the period of oscillations can be restricted to a narrow interval.

## Discussion

A better understanding of the molecular mechanisms underlying mammalian circadian rhythms will undoubtedly inform our efforts to improve human health and deal with modern societal problems such as shiftwork and jetlag. However, the inventory of genes and genetic interactions in the mammalian circadian-clock network is still incomplete. Important players may be yet unknown or under-appreciated [34, 35]. For example, recent experimental data about oscillations of an antisense RNA transcript in the circadian rhythm in mouse liver [14, 36, 37] suggest a possible antagonistic relationship between a core-clock mRNA, *Per2*, and its natural antisense partner, *Per2AS*. Because antisense transcripts can be fundamental regulators of gene expression, the interactions between *Per2* and *Per2AS* may be important factors for controlling circadian rhythms [1]. To date, the molecular mechanisms of *Per2-Per2AS* interactions are unknown. In this work, we propose two realistic mechanisms for these interactions and study their effects *in silico* by incorporating *Per2-Per2AS* interactions into a well-documented mathematical model [18] of mammalian circadian rhythms. In the first hypothesis, *Per2* mRNA molecules interfere with the transcription of *Per2AS* molecules and *vice versa*. In the second hypothesis, mature *Per2* and *Per2AS* molecules form double-stranded RNA duplexes, which are rapidly degraded by RNases.

Simulations and analysis of our *pre-transcriptional* model (the first hypothesis) show that mutual transcriptional interference can generate emergent oscillations in the clock network. That is to say, *Per2-Per2AS* interactions can generate new modes of circadian oscillations not seen in the original model [18]. For example (Fig 5; purple curve), our model predicts that *Per2AS* overexpression restores circadian rhythms to *ROR*-overexpressing cells by rebalancing the positive and negative interactions exerted on *BMAL1* expression by ROR and REV (Fig 1).

According to our *post-transcriptional* model (the second hypothesis), circadian oscillations are expected to be eradicated by an increasing rate of *Per2AS* expression, which is to be expected if *Per2AS* forms unstable duplex molecules with *Per2* mRNA. For both the *pre*- and *post-transcriptional* models and for a combined *pre/post*-model, we have computed how the period of oscillation and the amplitudes and phases of core clock gene oscillations will vary with the rate of synthesis of *Per2AS* transcripts (see Figs 4, 7 and 8). By altering the rate of expression of *Per2AS* transcripts, these predicted dependencies of period, amplitudes, and phases can be tested experimentally. Comparison between such experimental results and mathematical predictions can evaluate the accuracy and predictive power of the three alternative models of sense-antisense interactions. In this way, experimental interrogation, in combination with mathematical simulations, can shed light on the mechanisms of sense-antisense interactions in the mammalian circadian rhythm, and a more realistic mathematical model can be developed.

Of the three models we have studied (*pre-*, *post-*, and combined *pre/post-transcriptional* models), the *pre-transcriptional* model is the most likely, in our opinion, because it provides the most robust account of the observed, circadian, antiphasic oscillations of *Per2* and *Per2AS* RNAs [14], in the context of all the other experimental data that went into the development and parameterization of the circadian-rhythm model of Relogio *et al*. [18]. Furthermore, the *pre-transcriptional* model makes the counterintuitive prediction that *Per2AS* overexpression can restore circadian rhythms to cells that are overexpressing *ROR*. This striking prediction of the model can be tested in a suitably designed mutant strain of mouse liver cells that overexpress both *Per2AS* RNA and *Ror* mRNA.

Our study of sense-antisense interactions has been made in the context of a specific mathematical model of mammalian circadian rhythms [18], but we suspect that our results are generic, in the sense that similar results will be found if our hypotheses are tested in different models of the circadian clock [29–31, 38]. As an example, we studied the effects of *Per2* and *Per2AS* interactions in the Mirsky *et al*. model [30] of mammalian circadian rhythms. The three main differences between the Relogio and Mirsky models are that (a) Mirsky’s model includes paralogs of *Per* and *Cry* (i.e., *Per1* and *Per2*, *Cry1* and *Cry2*), (b) the two models make different assumptions about how PER/CRY interferes with CLOCK/BMAL-induced gene expression, and (c) *Rev* and *Ror* play less prominent roles in the generation of rhythmic dynamics in Mirsky’s model relative to Relogio’s model. Suppl. S11 Fig shows how period, amplitudes, and phases of oscillations change in the Mirsky *et al*. model [30] with increasing rate of *Per2AS* transcription. Notice the similarity between Fig 4 and Suppl. S11 Fig, despite the fact that Mirsky’s model distinguishes between *Per1* and *Per2* transcripts and proteins. Suppl. S12A Fig shows that *Per1* oscillations are indirectly affected by the double negative feedback interactions of *Per2* and *Per2AS*, but the amplitude changes of *Per1* and *Per2* are uncorrelated. Suppl. S12B Fig shows that *Cry1* and *Cry2* oscillations also respond to *Per2AS* interference, and that their amplitudes are anti-correlated with each other. The generic effects of *Per2*-*Per2AS* interactions in different models are due, presumably, to generic, network-level consequences of a double-negative feedback loop embedded in the delayed negative-feedback that generates circadian rhythms.

Obviously, depending on the choice of a base model, of the mathematical representations of our hypotheses, and of parameter values, a rich repertoire of interesting dynamics are possible in a mathematical model involving many feedback loops that can generate independent oscillations [39–41]. For example, in a recent paper El-Athman et al. [42] have combined the Relogio-2011 model of the mammalian circadian clock with a model of mammalian cell-cycle controls and shown that knocking out the tumor suppressors that bridge the two systems induces notable phase shifts in the expression of circadian clock genes. Interesting research directions in the future would be a) whether these phase shifts can be controlled by antisense transcripts of *Per2*, and b) whether the positive regulation of the tumor protein p53 by Per2, as reported by Gotoh et al. [43], can induce predictable amplitude and phase modulations in the oscillations of cell cycle elements. Finally, we hope that the modeling results reported here, suggesting that *Per2*-*Per2AS* interactions may have profound effects on circadian rhythmicity, may stimulate new experiments about the roles of this sense-antisense pair of RNAs in the mammalian circadian-clock network.

## Supporting information

S1 Text Derivation of the pre-transcriptional model (see Eqs (1A and 1B)) based on the molecular mechanism of transcriptional interference shown in Fig 1B and Suppl. S1 Fig. (DOCX)

S2 Text A minimal modification of Relogio et al. model of mammalian circadian rhythms to account for pre-transcriptional interactions between sense and antisense transcripts. A new term in the *Per* equation and an ODE for *Per2AS* are highlighted.
(DOCX)

S3 Text Adding new terms into Mirsky et al. model of mammalian circadian rhythms to account for pre-transcriptional interactions between *Per2* and *Per2AS*. (DOCX)

S4 Text Applying the ensemble method of parameter estimation for fitting the modified Relogio model to experimental data. (DOCX)

S5 Text Mathematica nb file for simulating of pre-transcriptional model. The code plots antiphasic dynamics of *Per2* and *Per2AS*, and calculates the period and phase difference between *Per2* and *Per2AS* oscillations.
(TXT)

S6 Text XPPAUT ode file for simulating the pre-transcriptional model. (TXT)

S1 Fig A wiring diagram of the molecular interactions between mature and nascent transcripts of *Per2* and *Per2AS*. (DOCX)

S2 Fig Comparisons of simulations of the ‘reduced’ (Eq (1)) and the ‘extended’ (Eqs (3–8) Suppl. S1 Text) versions of the *pre-transcriptional* model. (DOCX)

S3 Fig Time courses of *Per2* and *Per2AS* in simulations of exogenously overexpressed *Ror* strains. (DOCX)

S4 Fig Rescuing circadian rhythms by *Per2AS* overexpression in cells for which the oscillations were abolished by constitutive expression of REV-ERB. (DOCX)

S5 Fig **Circular plots of the phase distributions of core clock genes in (**A**) WT cells and (**B**) in cells that express a high level of REV-ERB and overexpress *Per2AS***.
(DOCX)

S6 Fig A diagram showing the domains of slow and emergent oscillations for the bifurcation parameters *λ* (*Per2AS* overexpression) and *x5*^0^ (constitutive REV-ERB expression). (DOCX)

S7 Fig Simulations of *Per2* and *Per2AS* rhythms in the post-transcriptional model. (DOCX)

S8 Fig Two-parameter bifurcation diagrams of the *post-transcriptional* model (Eq (2)). Chosen bifurcation parameter pairs are (*k*_assn,_ *λ*_0_) and (*k*_assn,_ *d*_dup_). The regions where *Per2* and *Per2AS* oscillations are circadian and antiphasic (see Eq (4)) are marked in the diagrams.
(DOCX)

S9 Fig Time courses of *Per2*, *Per2AS*, and *Dplx* in simulations of the post-transcriptional model (see Eq (2)) at different combinations of the parameters: *k*_assn_, *d*_dup_, and *λ*_0_. (DOCX)

S10 Fig Time courses of *Per2* and *Per2AS* in simulations of the combined model (see Eq (3)) at different values of *λ*. (DOCX)

S11 Fig Simulations of the modified Mirsky *et al*. model of the mammalian circadian clock. Period, amplitude, and phases of oscillations are plotted against *λ*, the rate of *Per2AS* expression.
(DOCX)

S12 Fig Comparisons of the dynamics of *Per1* vs *Per2*, and *Cry1* vs *Cry2* at different levels of *Per2AS* expression in simulations of the modified Mirsky *et al*. model (Suppl. S3 Text). (DOCX)

S1 Table Model parameter values. (DOCX)
