# Supplementary material for: Modeling the interactions of sense and antisense Period transcripts in the mammalian circadian clock network
Source: PLoS Comput Biol. 2018 Feb 15;14(2):e1005957. doi: 10.1371/journal.pcbi.1005957 (PMC5831635; doi:10.1371/journal.pcbi.1005957)
Supplement: S1 Text — (DOCX) [file pcbi.1005957.s001.docx]

**Supplementary Material to**

**Modeling the interactions of sense and antisense *Period* transcripts in the mammalian circadian clock network**

Dorjsuren Battogtokh, Shihoko Kojima, and John J. Tyson

**Suppl. Text S1. A molecular mechanism for the pre-transcriptional model of sense- and antisense-RNA interactions.**

In this subsection we propose molecular mechanisms for our *pre-transcriptional* model, Eqs. (1a-b) in the main text. We assume that, due to their complementary sequences, a mature *Per2* mRNA can form a duplex species with a nascent *Per2AS* RNA molecule that is being transcribed from the same DNA locus (see Fig. S1). We assume that duplex formation obstructs further transcription of the *Per2AS* RNA molecule and leads to immediate dissociation of the duplex from the DNA. The free duplex molecule disassociates into *Per2* mRNA and a short RNA fragment that, we assume, is unreactive and rapidly degraded into nucleoside monophosphates (NMPs):

*Per2* + *Per2AS*_nas_ → *Dplx*_AS_P_ → *Per2* + *Per2AS*_short_ → *Per2* + NMPs (1)

Similarly, we assume that mature *Per2AS* mRNAs form duplexes with nascent *Per2* transcripts and promote their degradation:

*Per2AS* + *Per2*_nas_ → *Dplx*_P_AS_ → *Per2AS* + *Per2*_short_ → *Per2AS* + NMPs (2)

The rate equations for the RNA species of the ‘extended’ model in Suppl. Fig. S1 are:

$\frac{d{[Per2AS}_{nas}]}{dt}=\alpha-\beta[Per2]{[Per2AS}_{nas}]-\gamma\left[ {Per2AS}_{nas} \right]-\sigma\left[ {Per2AS}_{nas} \right]$ (3)

$\frac{d[Per2AS]}{dt}=\sigma{[Per2AS}_{nas}]-\beta^{'}[Per2AS]{[Per2}_{nas}]+\eta'[{Dplx}_{P\_AS}]-\delta[Per2AS]$(4)

$\frac{d{[Dplx}_{P\_AS}]}{dt}=\beta^{'}[Per2AS]{[Per2}_{nas}]-\eta'[{Dplx}_{P\_AS}]$ (5)

$\frac{d{[Per2}_{nas}]}{dt}=\alpha'\cdot R(\ldots)-\beta'[Per2AS]{[Per2}_{nas}]-\gamma'\left[ {Per2}_{nas} \right]-\sigma'\left[ {Per2}_{nas} \right]$ (6)

$\frac{d[Per2]}{dt}=\sigma'{[Per2}_{nas}]-\beta[Per2]{[Per2AS}_{nas}]+\eta[{Dplx}_{AS\_P}]-\delta'[Per2]$ (7)

$\frac{d{[Dplx}_{AS\_P}]}{dt}=\beta[Per2]{[Per2AS}_{nas}]-\eta[{Dplx}_{AS\_P}]$ (8)

In Eqs. (3-8), the Greek letters (with or without primes) are rate constants: *α* is a zeroth-order rate constant (unit = nM h^−1^); *β* is a second-order rate constant (unit = nM^−1^ h^−1^); *γ*, *δ*, *η*, and *σ* are first-order rate constants (unit = h^−1^); *R*(…) is an order-one, dimensionless rate function, see Eq. (1c) of the main text, defined in the Relogio model [1].

To simplify the model, we make pseudo-steady state approximations on the concentrations of *Per2AS*_nas_, *Per2*_nas_, *Dplx*_P_AS_ and *Dplx*_AS_P_:

$\left[ {Per2AS}_{nas}^{0} \right]=\frac{\alpha}{\gamma+\sigma+\beta\left[ Per2 \right]}; \left[ {Per2}_{nas}^{0} \right]=\frac{\alpha^{'}\cdot R(\ldots)}{\gamma^{'}+\sigma^{'}+\beta^{'}\left[ Per2AS \right]} .$ (9)

$\left[ {Dplx}_{AS\_P}^{0} \right]=\frac{\beta}{\eta}\left[ {Per2AS}_{nas} \right][Per2]; \left[ {Dplx}_{P\_AS}^{0} \right]=\frac{\beta^{'}}{\eta^{'}}\left[ {Per2}_{nas} \right][Per2AS] .$ (10)

Provided that *σ, δ* << *γ*, *η*, $\sqrt{\alpha\beta}$, and similarly for the primed parameters, the ‘slow’ variables in this approximation are

$\left[ Per2 \right]_{T}=\left[ Per2 \right]+\left[ {Dplx}_{AS\_P} \right]=[Per2]\left( 1+\frac{\beta}{\eta}[{Per2AS}_{nas}] \right)$ (11)

$\left[ Per2AS \right]_{T}=\left[ Per2AS \right]+\left[ {Dplx}_{P\_AS} \right]=[Per2AS]\left( 1+\frac{\beta^{'}}{\eta^{'}}[{Per2}_{nas}] \right)$ (12)

which are governed by the ‘slow’ differential equations:

$\frac{d\left[ Per2AS \right]_{T}}{dt}=\sigma\left[ {Per2AS}_{nas} \right]-\delta[Per2AS]=\frac{\lambda K_{S}}{K_{S}+\left[ Per2 \right]}-\delta[Per2AS]$ (13)

$\frac{d{[Per2]}_{T}}{dt}=\sigma^{'}\left[ {Per2}_{nas} \right]-\delta^{'}[Per2]=a\cdot V_{1max}R(\ldots)\frac{\mu K_{AS}}{K_{AS}+\left[ Per2AS \right]}-\delta^{'}[Per2]$ (14)

where *λ* = *ασ*/(*γ*+*σ*), *K*_S_ = (*γ*+*σ*)/*β*, *μ∙a∙V*_1max_ = *α'σ'*/(*γ'*+*σ'*), and *K*_AS_ = (*γ'*+*σ'*)/*β'*. In Eq. (14) we have introduced the parameters *a* and *V*_1max_ from the differential equation for *Per2* mRNA in Relogio’s model, where [*Per2*] is called ‘*y1*’. Notice that, in Eqs. (13) and (14), the units of *λ* and *μ∙a∙V*_1max_ are nM h^−1^, of *K*_S_ and *K*_AS_ are nM, of *δ* and *δ'* are h^−1^, and *R* is dimensionless. Finally, notice that the parameters *δ* and *δ'* in this subsection are called, respectively, *d_AS_* and *d_Per2_* in the main text, and the parameter we call *d_Per2_* is called *d_y1_* in Relogio’s model.

Equations (13) and (14) are the ODEs of the ‘reduced’ model that we wanted to derive, *i.e*., Eqs. (1a) and (1b) in the main text, provided that [*Per2*] ≈ [*Per2*]_T_ and [*Per2AS*] ≈ [*Per2AS*]_T_; i.e., provided

$\left[ {Per2AS}_{nas}^{0} \right]=\frac{\alpha}{\gamma+\sigma+\beta\left[ Per2 \right]}\ll\frac{\eta}{\beta} ; \left[ {Per2}_{nas}^{0} \right]=\frac{\alpha^{'}\cdot R\left( \ldots\right)}{\gamma^{'}+\sigma^{'}+\beta^{'}\left[ Per2AS \right]}\ll\frac{\eta^{'}}{\beta^{'}} .$ (15)

We can write inequality (15A) as

$\frac{\alpha}{K_{S}}\cdot\frac{K_{S}}{K_{S}+\left[ Per2 \right]}<\frac{\alpha}{K_{S}}\ll\eta$ ; (16)

and, recalling that *R*(…) = O(1), we can write a similar requirement for inequality (15B). Hence, the conditions for deriving Eqs. (1a-1b) from the mechanism in Suppl. Fig. S1 are that

*σ, δ* << *γ*, *αβ*/*γ*  << *η*. (17A)

*σ', δ'* << *γ'*, *α'β'*/*γ'*  << *η'*. (17B)

The following set of parameter values satisfies these inequalities:

*α* = 10, *β* = 200, *γ* = 18, *δ* = 2, *η* = 1000, *σ* = 2. (18A)

*α'* = 120, *β'* = 3, *γ'* = 2.7, *δ'* = 0.3, *η'* = 100, *σ'* = 0.3. (18B)

and maintains consistency with the WT values specified in Table 1 of the main text; namely,

*λ* = 1, *K*_AS_ = 1, *μ* = 1, *K*_S_ = 0.1, *a* = 12, *V*_1max_ = 1. (19)

In Suppl. Fig. S2 we compare simulations of the ‘reduced’ model (Relogio’s differential equations, supplemented with Eqs. (13)-(14); see Suppl. Text S2) with simulations of the ‘extended’ model (Relogio’s differential equations, supplemented with Eqs. (3)-(8)), for WT parameter values (Table 1) and ‘extended’ parameter values given in Eq. (18). Clearly the reduced and extended versions of Relogio’s model give very similar results, provided inequalities (17) are satisfied.

[1]. Relógio A, Westermark PO, Wallach T, Schellenberg K, Kramer A, Herzel H. Tuning the Mammalian Circadian Clock: Robust Synergy of Two Loops. *PLOS Comput. Biol.* **7**:e1002309 (2011).
