# Supplementary material for: Modeling the interactions of sense and antisense Period transcripts in the mammalian circadian clock network
Source: PLoS Comput Biol. 2018 Feb 15;14(2):e1005957. doi: 10.1371/journal.pcbi.1005957 (PMC5831635; doi:10.1371/journal.pcbi.1005957)
Supplement: S2 Text — A new term in the Per equation and an ODE for Per2AS are highlighted. (DOCX) [file pcbi.1005957.s002.docx]

**Suppl. Text S2. Differential equations of the pre-transcriptional model of mammalian circadian rhythms, based on Relogio *et al.* [1].**

CLOCK/BMAL

 (1)

*Rev-Erb*

 (2)

*Ror*

 (3)

REV-ERB_C_

 (4)

ROR_C_

 (5)

REV-ERB_N_

 (6)

ROR_N_

 (7)

*Bmal*

 (8)

BMAL_C_

 (9)

BMAL_N_

 (10)

*Per2*

$$\frac{dy1}{dt}=V_{1max}\frac{1+a\left( \frac{x1}{k_{t1}} \right)^{b}}{1+{\left( \frac{PC}{k_{i1}} \right)^{c}\left( \frac{x1}{k_{t1}} \right)}^{b}+\left( \frac{x1}{k_{t1}} \right)^{b}}\frac{\boldsymbol{\mu}\boldsymbol{K}_{\boldsymbol{AS}}}{\boldsymbol{K}_{\boldsymbol{AS}}\boldsymbol{+AS}}-d_{y1}y1 (11)$$

*Cry*

 (12)

CRY_C_

1

3

1

*z*2

1·.

5

4

)

2

2

(

1

1

4

5

5

4

0

2

*z*

*d*

*z*

*z*

*kf*

*z*

*kf*

*z*

*kd*

*z*

*kd*

*y*

*y*

*k*

*dt*

*dz*

*z*

*z*

*z*

*z*

*z*

*p*

−

−

−

+

+

+

=

(13)

PER_C_

 (14)

PER_C_^*^

 (15)

PER_C_^*^/CRY_C_

 (16)

PER_C_/CRY_C_

 (17)

PER^*^_N_/CRY_N_

 (18)

PER_N_/CRY_N_

 (19)

PER/CRY_pool_

*PC* = *x*2 + *x*3 (20)

*Per2AS*

$\frac{dAS}{dt}=\frac{\lambda K_{S}}{K_{S}+y1}-d_{AS}AS$ (21)
