# Supplementary material for: Modeling the interactions of sense and antisense Period transcripts in the mammalian circadian clock network
Source: PLoS Comput Biol. 2018 Feb 15;14(2):e1005957. doi: 10.1371/journal.pcbi.1005957 (PMC5831635; doi:10.1371/journal.pcbi.1005957)
Supplement: S3 Text — (DOCX) [file pcbi.1005957.s003.docx]

**Suppl. Text S3. Adding *Per2AS* to the Mirsky *et al*. model.**

To determine whether the effects of sense (*Per2*) and antisense (*Per2AS*) interactions that we observed in Relogio’s model are common to other models of circadian rhythms, we introduced our *pre-transcriptional* hypothesis into a model of the mammalian circadian rhythm due to Mirsky *et al.* [1]. First, to the differential equation for *Per2*, we introduced an additional factor for *Per2AS* repression of *Per2* transcription, highlighted in red,

$\frac{dPer2}{dt}=\alpha_{mirsky\_Per2}\frac{\mu K_{AS}}{K_{AS}+Per2AS}-k_{mPer2}Per2.$

The function $\alpha_{mirsky\_Per2}$ represents the term in the original model describing transcriptional regulation of the *Per2* gene. It depends on several other variables and parameters in the model. Second, we extended the model by a differential equation describing *Per2AS* RNA synthesis and degradation,

$\frac{dPer2AS}{dt}=\frac{\lambda K_{S}}{K_{S}+y1}-d_{AS}Per2AS$.

As a result, the modified Mirsky model includes 22 variables and 137 parameters. In our simulations (Suppl. Figure S11- S12), we fixed the parameter values describing sense-antisense interactions at $\mu=1, K_{S}=0.05, K_{AS}=1$ and $d_{AS}=2$, while using $\lambda$ as the control parameter. All other parameters are fixed at the values chosen by Mirsky *et al*. [1].

[1]. Mirsky HP, Liu AC, Welsh DK, Kay SA, Doyle FJ. A model of the cell-autonomous mammalian circadian clock. *Proc. Natl. Acad. Sci. USA* **106**:11107-12 (2009).
