# Supplementary material for: Modeling the interactions of sense and antisense Period transcripts in the mammalian circadian clock network
Source: PLoS Comput Biol. 2018 Feb 15;14(2):e1005957. doi: 10.1371/journal.pcbi.1005957 (PMC5831635; doi:10.1371/journal.pcbi.1005957)
Supplement: S4 Text — (DOCX) [file pcbi.1005957.s004.docx]

**Suppl. Text S4. Fitting data using the ensemble method.**

The ensemble method was previously used for parameter estimation of a mathematical model of the circadian rhythm in *Neurospora* [1]. The ensemble method was studied in an integrated workflow of different parameter estimation methods in [2]. Briefly, the ensemble method is based on the Markov-Chain Monte-Carlo technique, using Metropolis-Hastings sampling to identify parameter values in mathematical models described by a system ODE’s representing a complex biochemical reaction network. In situations when a mathematical model is parameter rich but the quantity and quality of experimental data is limited, the goal of the ensemble method is to identify an ensemble of parameter sets consistent with, and constrained by, the available experimental data, instead of trying to identify the global minimum of the object function for the best fit. Because of the uncertainties and errors inherent in experiments (e.g. high-throughput data), the ensemble method emphasizes building data structures and computing statistical properties of the parameter ensemble.

The ensemble of parameter sets for Relogio *et al.* model is obtained by minimizing a multi-objective cost function [3],

$C=\theta_{expr}\sum_{i=1}^{\#genes(N)} {(E_{i}-E_{i}^{exp})}^{2}+\theta_{phase}\sum_{i=1}^{N} {(\varphi_{i}-\varphi_{i}^{exp})}^{2}+\theta_{mut}\sum_{i=1}^{N} {(\delta_{i}-\delta_{i}^{exp})}^{2},$

where $\theta_{expr}, \theta_{phase},$ and $\theta_{mut}$ are the weights assigned to the experimental data for the expression levels, for known phase distributions of core clock genes, and for knock-out and overexpressed phenotypes. $E_{i}^{exp}, \varphi_{i}^{exp}$ and $\delta_{i}^{exp}$ are experimental data about expression levels, phases and knock-out and overexpressed phenotypes (reported by Koike *et al.* [4] and Relogio *et al.* [5]). $E_{i}, \varphi_{i}$ and $\delta_{i}$ are the variables simulated by Relogio’s model. We used random initial estimates of all model parameters.

Parameter Sets I, II and III reported in Suppl. Table S1 are representatives of the best-fitting parameter sets. The values of the cost function for parameter sets I-III are about the same.

**References**

[1]. Yu Y, Dong W, Altimus C, Tang X et al., A genetic network for the clock of *Neurospora crassa*. *Proc. Natl. Acad. Sci. USA* **104**:2809–14 (2007).

[2]. Gome-Cbrero D, Compte A, Tegner J. Workflow for generating competing hypothesis from models with parameter uncertainty. *Interface Focus* **1**:438–449 (2011).

[3]. Transtrum MK, Machta BB, Sethna JP. Geometry of nonlinear least squares with applications to sloppy models and optimization. *Phys. Rev. E* **83**:036701 (2011).

[4]. Relógio A, Westermark PO, Wallach T, Schellenberg K, Kramer A, Herzel H. Tuning the Mammalian Circadian Clock: Robust Synergy of Two Loops. *PLOS Comput. Biol.* **7**:e1002309 (2011).

[5]. Koike N, Yoo S-H, Huang H-C, Kumar V, Lee C, Kim T-K, et al. Transcriptional Architecture and Chromatin Landscape of the Core Circadian Clock in Mammals. Science (New York, NY). 2012;338(6105):349-54. doi: 10.1126/science.1226339. PubMed PMID: PMC3694775.
