# Supplementary material for: Modeling the interactions of sense and antisense Period transcripts in the mammalian circadian clock network
Source: PLoS Comput Biol. 2018 Feb 15;14(2):e1005957. doi: 10.1371/journal.pcbi.1005957 (PMC5831635; doi:10.1371/journal.pcbi.1005957)
Supplement: S1 Fig — (DOCX) [file pcbi.1005957.s007.docx]

|  |
| --- |
| **Suppl. Figure S1.** Extended mechanism for reactions (**top**) between mature *Per2* transcripts and nascent *Per2AS* transcripts to form duplex RNA (*DplxAS_P*) and (**bottom**) between mature *Per2AS* transcripts and nascent *Per2* transcripts to form duplex RNA (*DplxP_AS*). The Greek letters indicate the rate constants for the individual reactions. NMP = nucleoside monophosphate pool; NTP = nucleoside triphosphate pool. |
