# Supplementary material for: Modeling the interactions of sense and antisense Period transcripts in the mammalian circadian clock network
Source: PLoS Comput Biol. 2018 Feb 15;14(2):e1005957. doi: 10.1371/journal.pcbi.1005957 (PMC5831635; doi:10.1371/journal.pcbi.1005957)
Supplement: S2 Fig — (DOCX) [file pcbi.1005957.s008.docx]

**
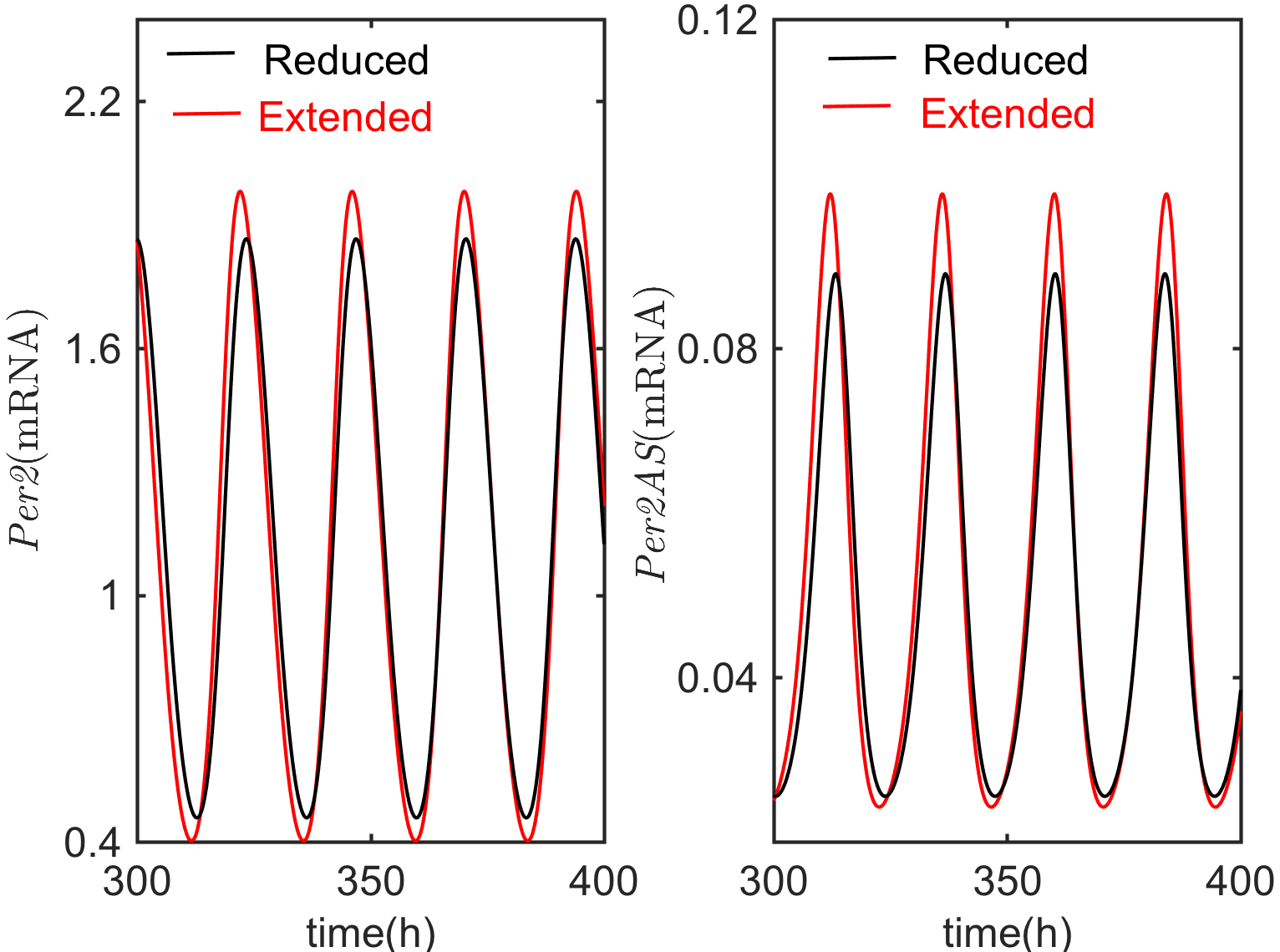
**

**Suppl. Figure S2.** Simulations of *Per2* and *Per2AS* oscillations in the ‘reduced’ and ‘extended’ versions of the *pre-transcriptional* model. The ‘reduced’ model consists of the differential equations in Suppl. Text S2, with WT parameter values from Suppl. Table 1. The ‘extended’ version supplements Relogio’s differential equations with Eqs. (3)-(8) of Suppl. Text S1 and supplementary parameter values given in Eq. (18) of Suppl. Text S1. Notice that simulations of the reduced model with WT parameter values provided here are very similar to simulations of the reduced model with Parameter Set III provided in Figure 2 of the main text.
