# Supplementary material for: Modeling the interactions of sense and antisense Period transcripts in the mammalian circadian clock network
Source: PLoS Comput Biol. 2018 Feb 15;14(2):e1005957. doi: 10.1371/journal.pcbi.1005957 (PMC5831635; doi:10.1371/journal.pcbi.1005957)
Supplement: S3 Fig — (DOCX) [file pcbi.1005957.s009.docx]

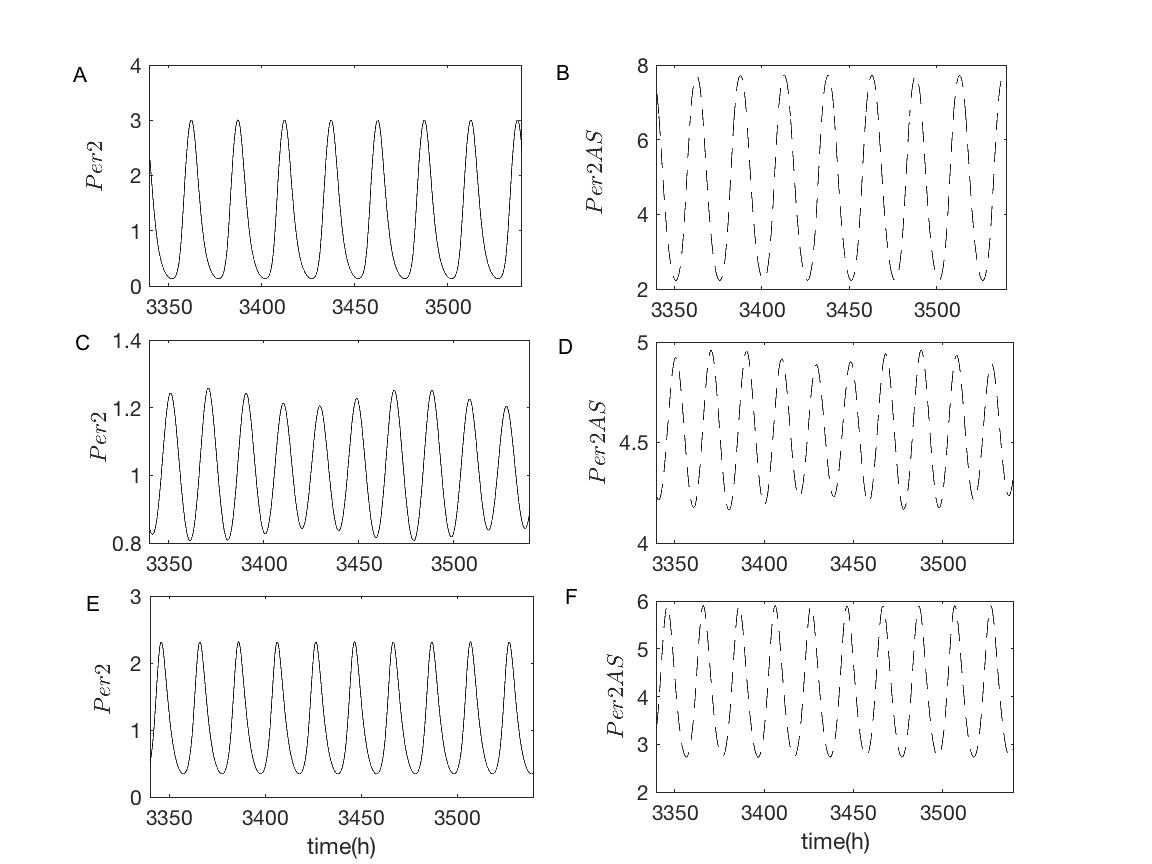


**Suppl. Figure S3.** Time courses of *Per2* and *Per2AS* for exogenously overexpressed *Ror.* Parameter values are *μ* =1.6, *λ* = 20. Other parameters are the same as in WT Relogio parameters. **(A, B)** *y4*_0_ = 1. **(C, D)** *y4*_0_ = 8. **(E, F)** *y4*_0_ = 20.
