# Supplementary material for: Modeling the interactions of sense and antisense Period transcripts in the mammalian circadian clock network
Source: PLoS Comput Biol. 2018 Feb 15;14(2):e1005957. doi: 10.1371/journal.pcbi.1005957 (PMC5831635; doi:10.1371/journal.pcbi.1005957)
Supplement: S4 Fig — (DOCX) [file pcbi.1005957.s010.docx]

**
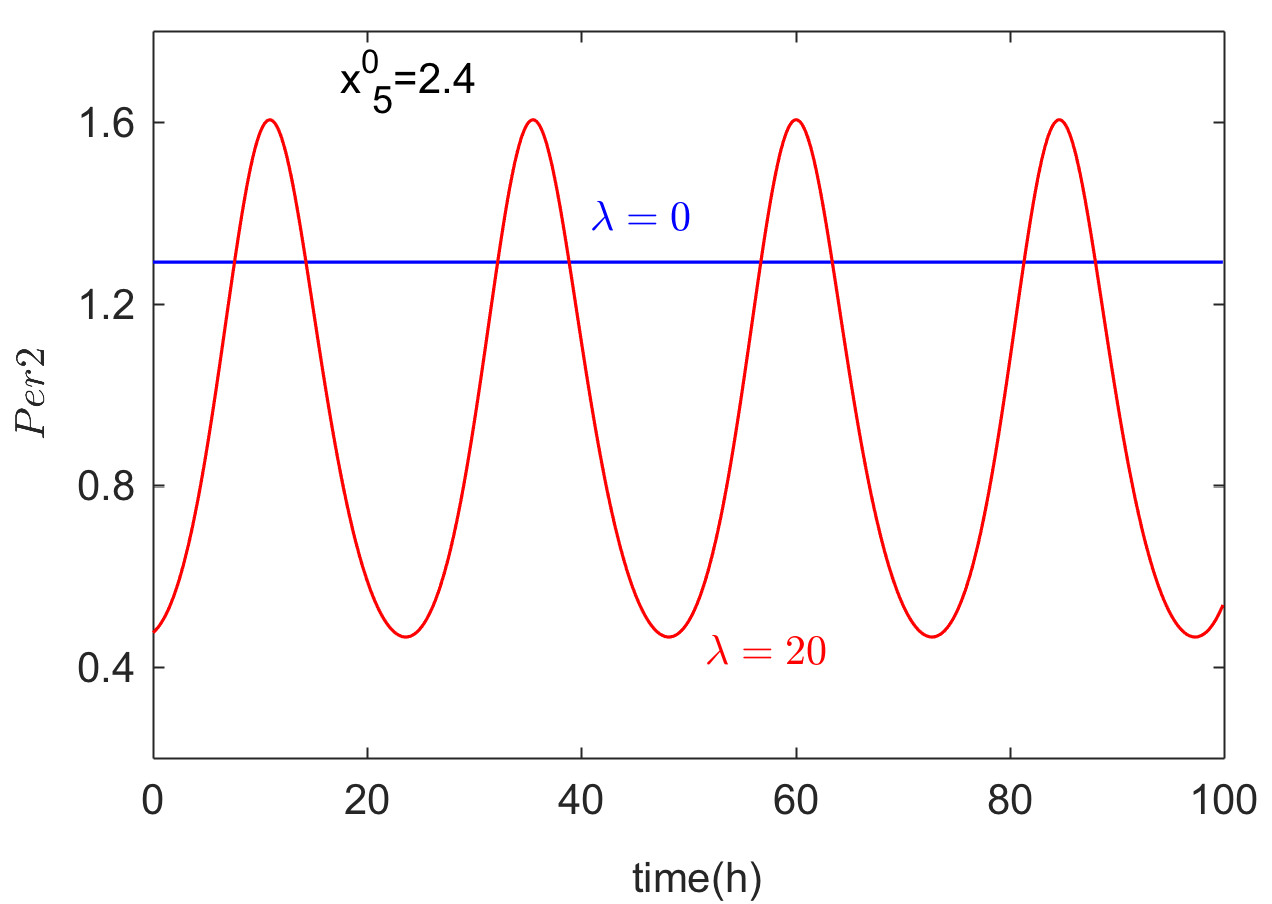
**

**Suppl. Figure S4.** Overexpression of *Per2AS* restores circadian oscillations in cells for which the oscillations were abolished by constitutive expression of REV (*x5*^0^ = [REV-ERB_N_] = 2.4). Blue curve: no *Per2AS* (*λ* = 0). Red curve: overexpression of *Per2AS* (*λ* = 20).
