# Supplementary material for: Modeling the interactions of sense and antisense Period transcripts in the mammalian circadian clock network
Source: PLoS Comput Biol. 2018 Feb 15;14(2):e1005957. doi: 10.1371/journal.pcbi.1005957 (PMC5831635; doi:10.1371/journal.pcbi.1005957)
Supplement: S5 Fig — Circular plots of the phase distributions of core clock genes in (A) WT cells and (B) in cells that express a high level of REV-ERB and overexpress Per2AS. (DOCX) [file pcbi.1005957.s011.docx]

**
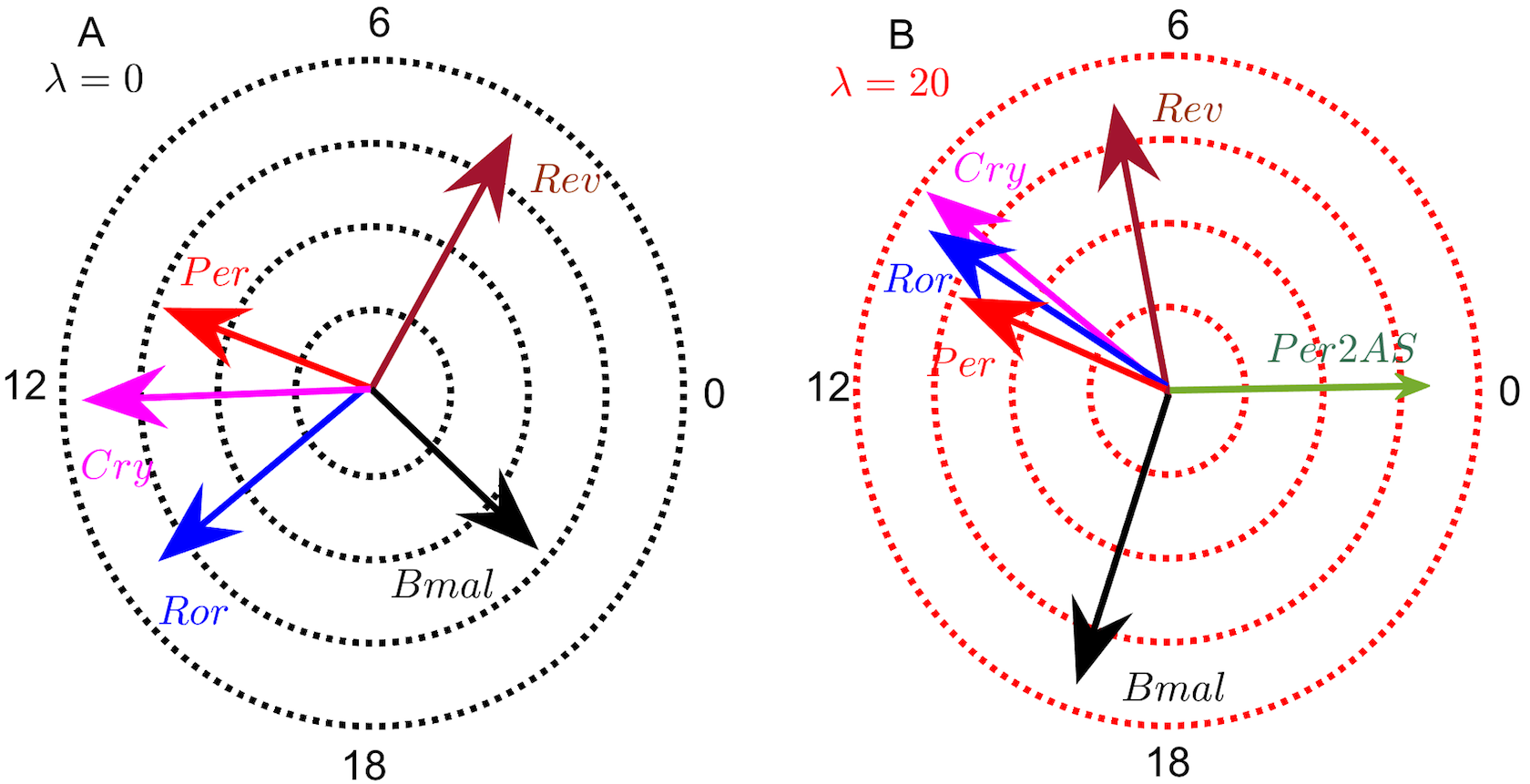
**

**Suppl. Figure S5.** The distributions of the phases of core clock genes in (**A**) WT cells and (**B**) in cells that express a high level of nuclear REV-ERB and overexpress *Per2AS* (see Suppl. Figure S4, *λ* = 20). The length of each vector corresponds to the expression level of the gene, and the angle corresponds to its phase of maximum amplitude of expression, relative to *Per2AS* (phase angle = 0). Notice that, in *Per2AS* overexpressing cells, the phases of *Per*, *Cry*, and *Ror* are locked at the same value (Figure 6).
