# Supplementary material for: Modeling the interactions of sense and antisense Period transcripts in the mammalian circadian clock network
Source: PLoS Comput Biol. 2018 Feb 15;14(2):e1005957. doi: 10.1371/journal.pcbi.1005957 (PMC5831635; doi:10.1371/journal.pcbi.1005957)
Supplement: S6 Fig — (DOCX) [file pcbi.1005957.s012.docx]

**
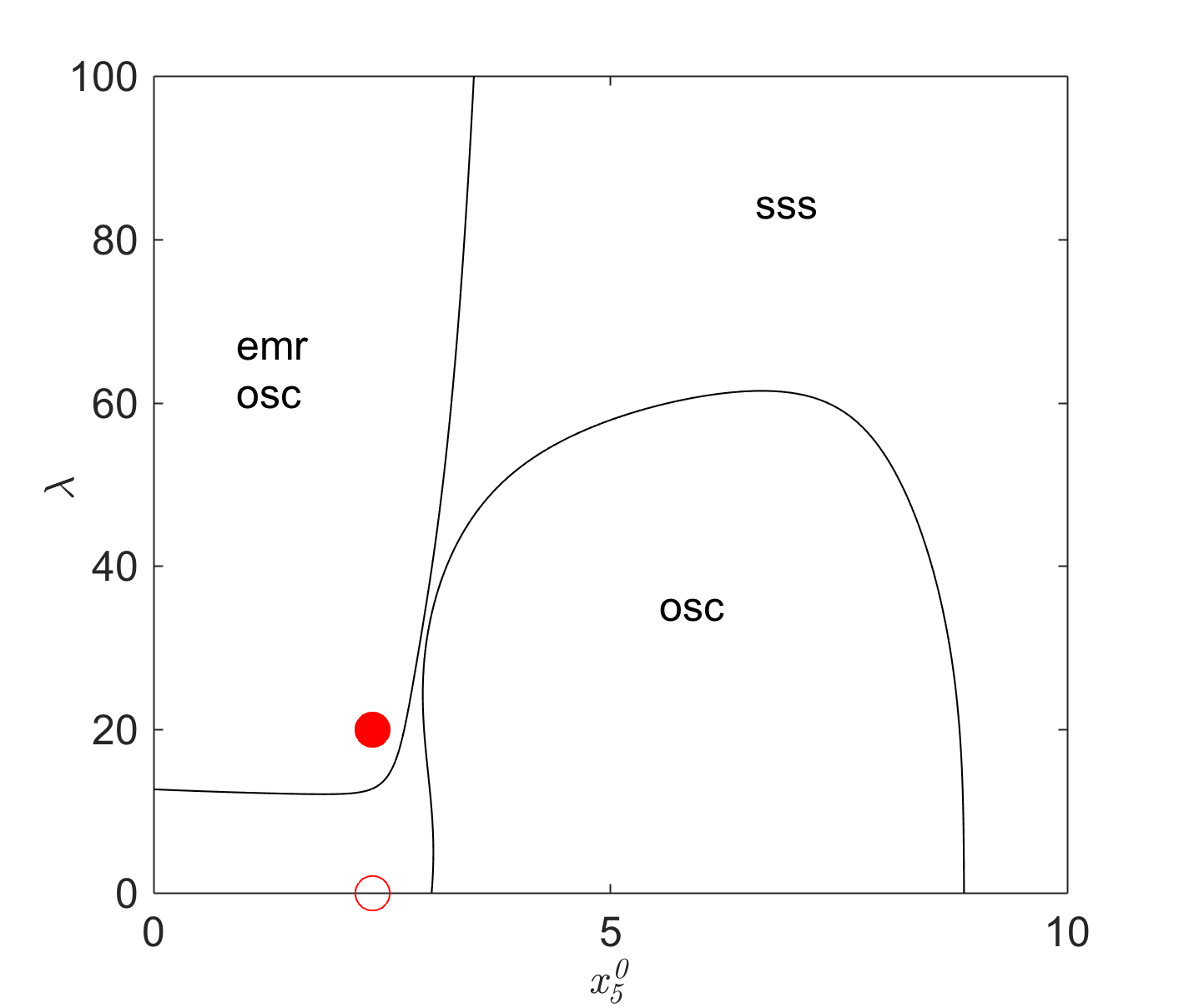
**

**Suppl. Figure S6.** A two-parameter bifurcation diagram of a model with constitutively expressed *REV* using *x5*^0^ (nuclear protein level of REV-ERB) and *λ* (transcription rate of *Per2AS*) as bifurcation parameters. Red open circle marks the steady state solution of Relogio’s model at *x5*^0^ = 2.4 (see Suppl. Figure S4, blue curve). Slow oscillations appear when *x5*^0^ is increased further; the period of oscillations in the right domain (*x5*^0^ > 3) is ~32 h. Closed red circle marks emergent circadian oscillations found in the left domain, for *λ* =20 (compare Suppl. Figure S4, red curve).
