# Supplementary material for: Modeling the interactions of sense and antisense Period transcripts in the mammalian circadian clock network
Source: PLoS Comput Biol. 2018 Feb 15;14(2):e1005957. doi: 10.1371/journal.pcbi.1005957 (PMC5831635; doi:10.1371/journal.pcbi.1005957)
Supplement: S7 Fig — (DOCX) [file pcbi.1005957.s013.docx]

**
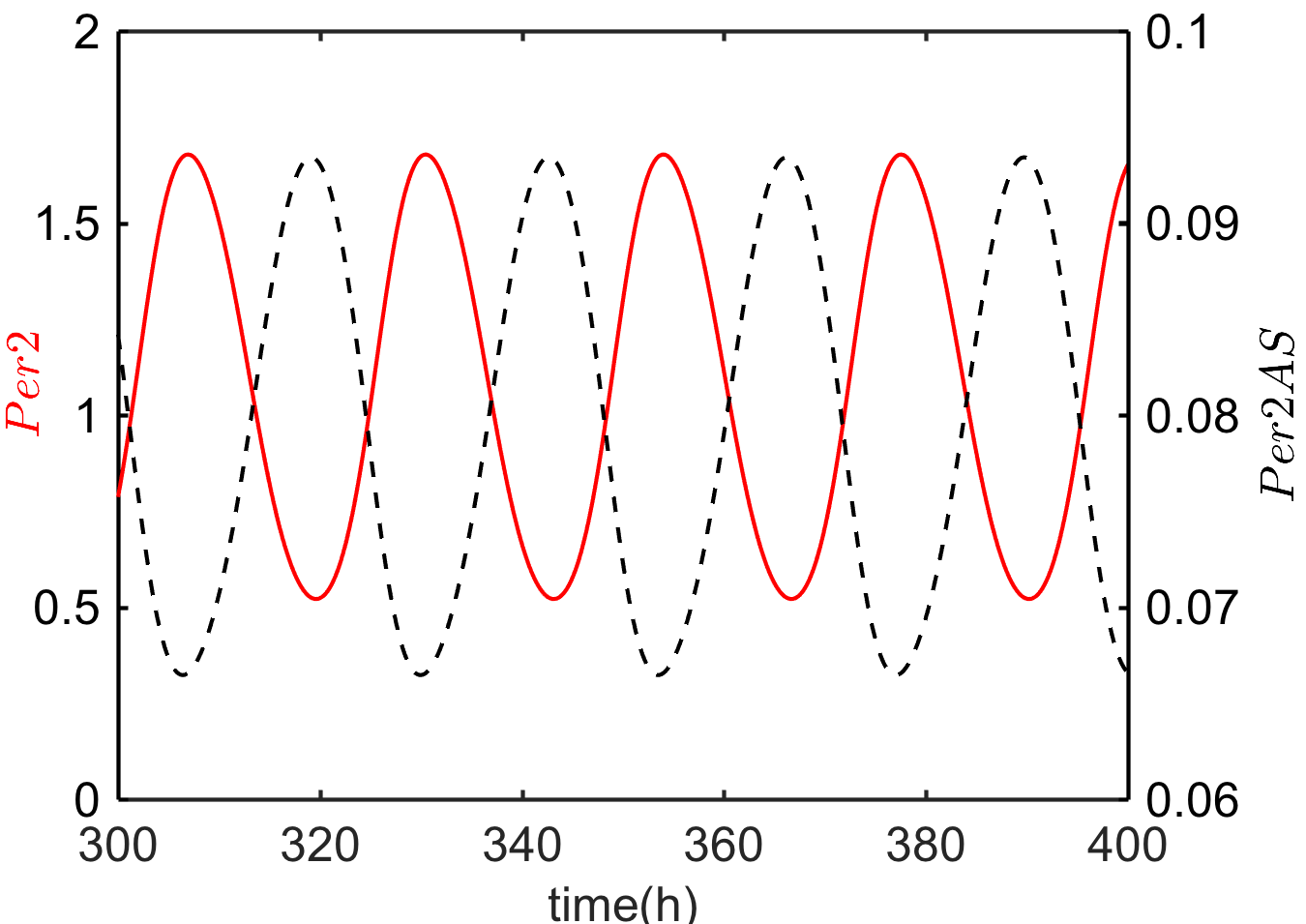
**

**Suppl. Figure S7.** Time courses of *Per2* and *Per2AS* in the post-transcriptional model. *λ*_0_ = 0.2, *k*_assn_ = 1 and *d*_dup_ = 0.1. Other parameters are at the same values as WT parameters.
