# Supplementary material for: Modeling the interactions of sense and antisense Period transcripts in the mammalian circadian clock network
Source: PLoS Comput Biol. 2018 Feb 15;14(2):e1005957. doi: 10.1371/journal.pcbi.1005957 (PMC5831635; doi:10.1371/journal.pcbi.1005957)
Supplement: S8 Fig — Chosen bifurcation parameter pairs are (kassn, λ0) and (kassn, ddup). The regions where Per2 and Per2AS oscillations are circadian and antiphasic (see Eq (4)) are marked in the diagrams. (DOCX) [file pcbi.1005957.s014.docx]

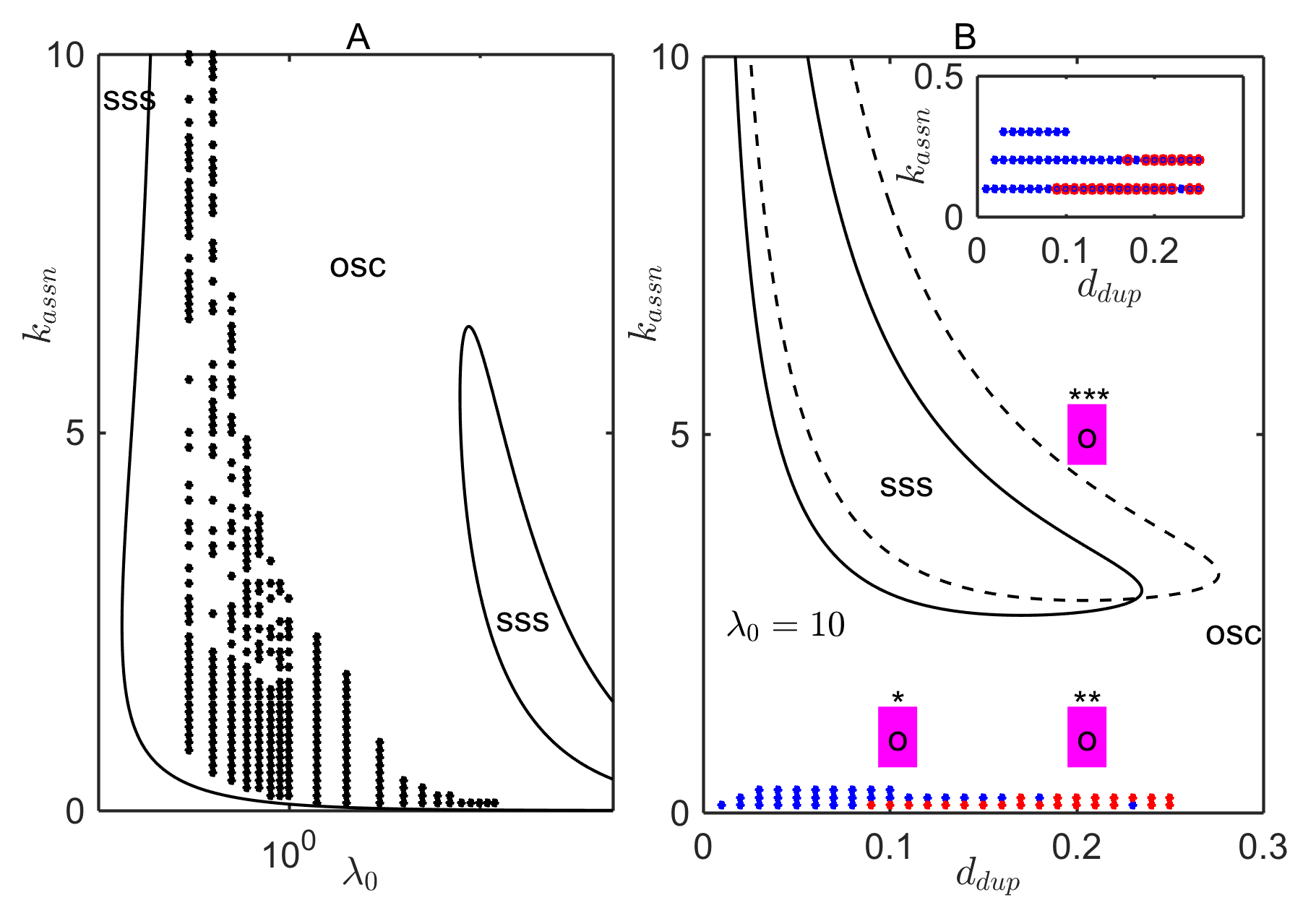


**Suppl. Figure S8.** Two-parameter bifurcation diagram for the *post-transcriptional* model. (**A**) Solid lines show loci of Hopf bifurcation points. Small black symbols mark regions where conditions:

$23 \text{h} <T< 25 \text{h},$ (S8.1)

($A_{max}^{Per2}-A_{min}^{Per2})>0.5$, (S8.2)

${11 \text{h}<|\phi}_{Per2}-\phi_{Per2AS}|<13 \text{h; }\phi_{X}=t(X=X_{max})$, (S8.3)

are fulfilled. Other parameter values are *μ*= 1.693, *k*_diss_ = 0.1, *d*_dup_ = 0.1; and as in the Relogio model. (**B**) Solid and dashed black lines show the loci of Hopf bifurcation points for *k*_diss_ = 0.1 and *k*_diss_ = 0.15, respectively. Small red symbols mark the region where conditions (S8.3) are fulfilled. The blue symbols mark the region where the (S8.3) is 9 h < |ϕ_Per2_ − ϕ_Per2AS_| < 15 h. The red and blue symbols were obtained for *k*_diss_ = 0.1*.* Purple and star(*) symbols mark parameter values used for the simulations in Suppl. Figure S9. Parameters of Relogio’s model are WT values; other parameter values are *μ* = 1.693 and *λ*_0_ = 10. The inset plots the figure in the range of small *k*_assn_.
