# Supplementary material for: Modeling the interactions of sense and antisense Period transcripts in the mammalian circadian clock network
Source: PLoS Comput Biol. 2018 Feb 15;14(2):e1005957. doi: 10.1371/journal.pcbi.1005957 (PMC5831635; doi:10.1371/journal.pcbi.1005957)
Supplement: S9 Fig — (DOCX) [file pcbi.1005957.s015.docx]

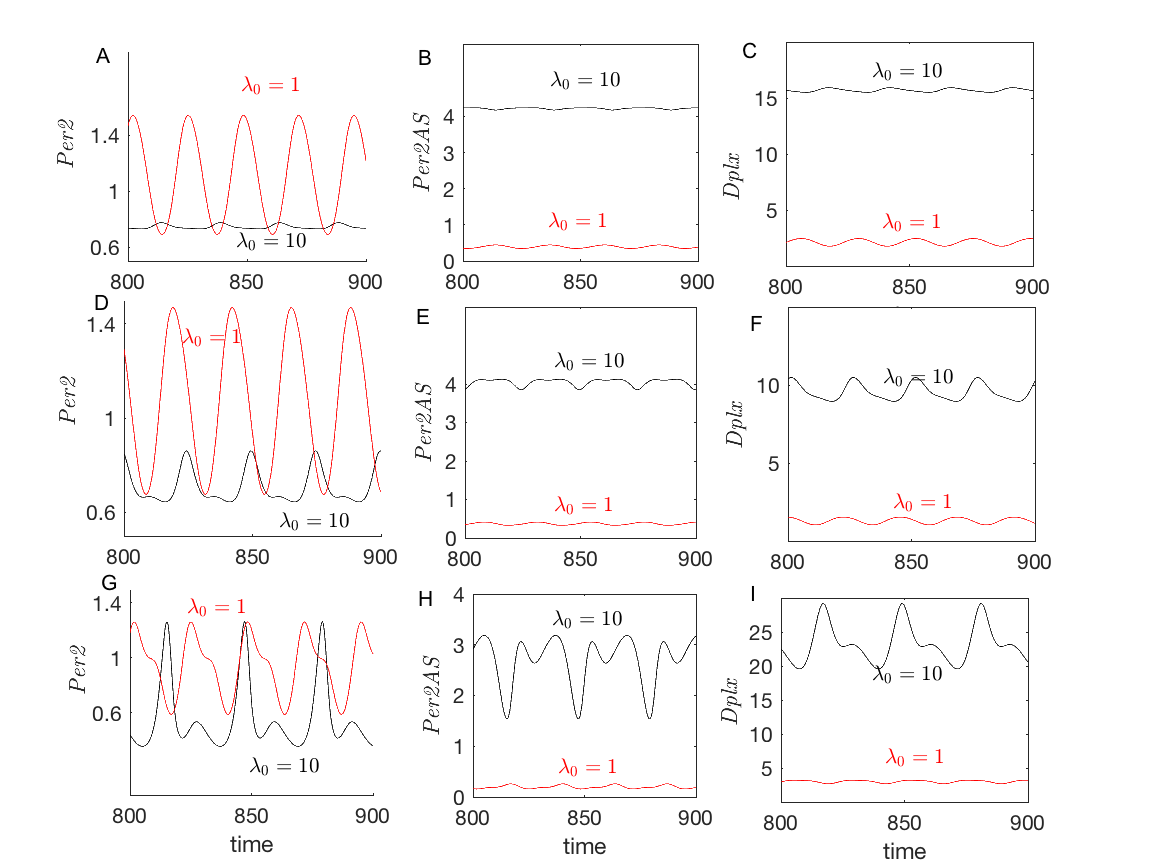


**Suppl. Figure S9.** Time courses of *Per2, Per2AS*, and *Dplx* in the post-transcriptional model. **(A-C)** *k*_assn_ = 1, *d*_dup_ = 0.1 (the point marked by * in Suppl. Figure 8B). With the increase of *λ*_0_, the amplitude of *Per2* oscillations dampens considerably. **(D-F)** *k*_assn_ = 1, *d*_dup_ = 0.2 1 (the point marked by ** in Suppl. Figure 8B). *Per2* oscillations become non-harmonic with the increase of *Per2AS* expression. **(G-I)** *k*_assn_ = 5, *d*_dup_ = 0.2 (the point marked by *** in Suppl. Figure 8B). The amplitude of *Per2* oscillations increases with the increase of *k*_assn_, but the time-course of *Per2* oscillations becomes strongly non-harmonic.
