# Supplementary material for: Modeling the interactions of sense and antisense Period transcripts in the mammalian circadian clock network
Source: PLoS Comput Biol. 2018 Feb 15;14(2):e1005957. doi: 10.1371/journal.pcbi.1005957 (PMC5831635; doi:10.1371/journal.pcbi.1005957)
Supplement: S10 Fig — (DOCX) [file pcbi.1005957.s016.docx]

**
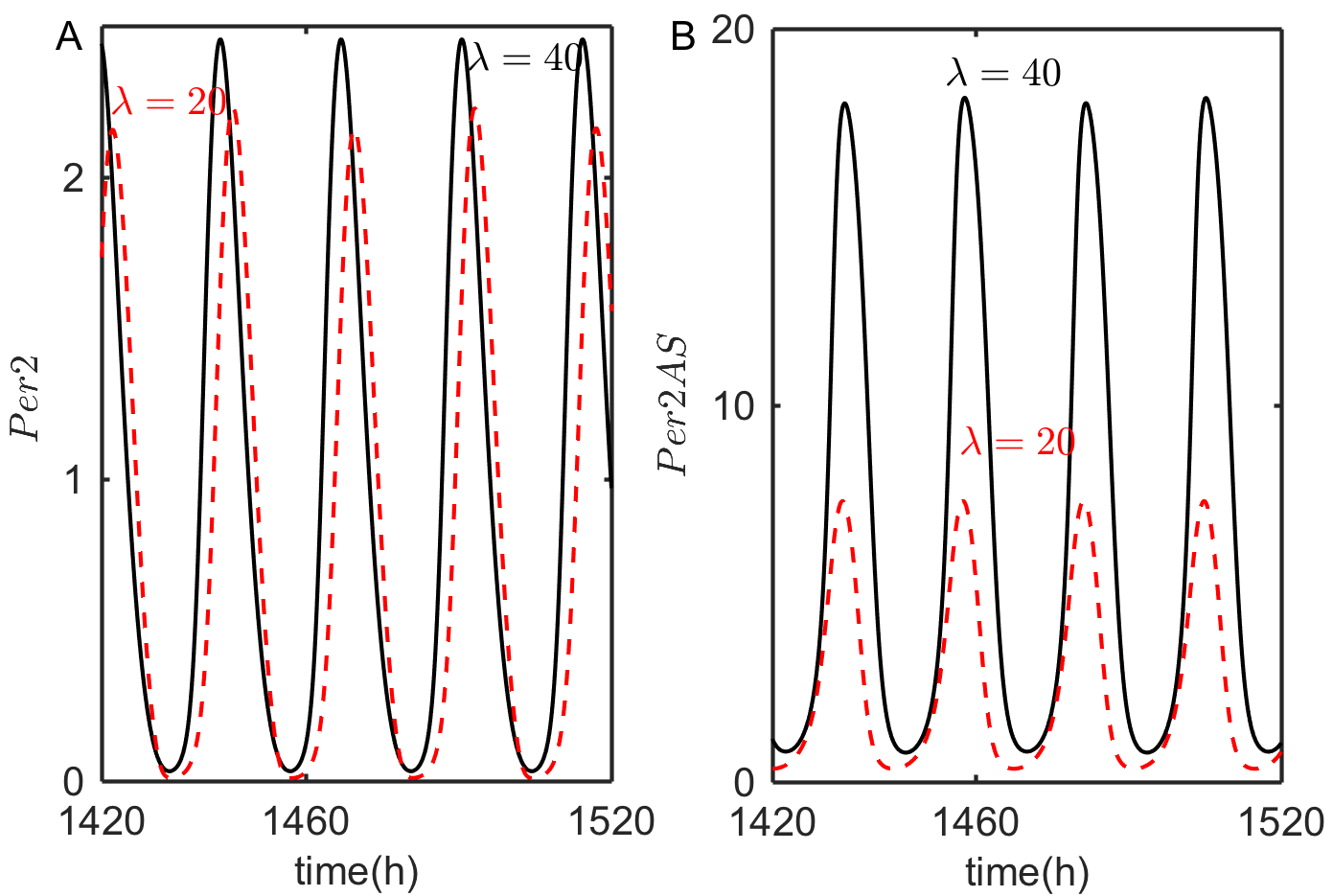
**

**Suppl. Figure S10.** Time courses of *Per2* and *Per2AS* in the combined model. Notice that, as *λ* increases from 20 (solid line) to 40 (dashed line), although the amplitude of *Per2AS* oscillations changes by more than two-fold, neither the amplitude nor the period of *Per2* oscillations changes significantly.
