# Supplementary material for: Modeling the interactions of sense and antisense Period transcripts in the mammalian circadian clock network
Source: PLoS Comput Biol. 2018 Feb 15;14(2):e1005957. doi: 10.1371/journal.pcbi.1005957 (PMC5831635; doi:10.1371/journal.pcbi.1005957)
Supplement: S11 Fig — Period, amplitude, and phases of oscillations are plotted against λ, the rate of Per2AS expression. (DOCX) [file pcbi.1005957.s017.docx]

**
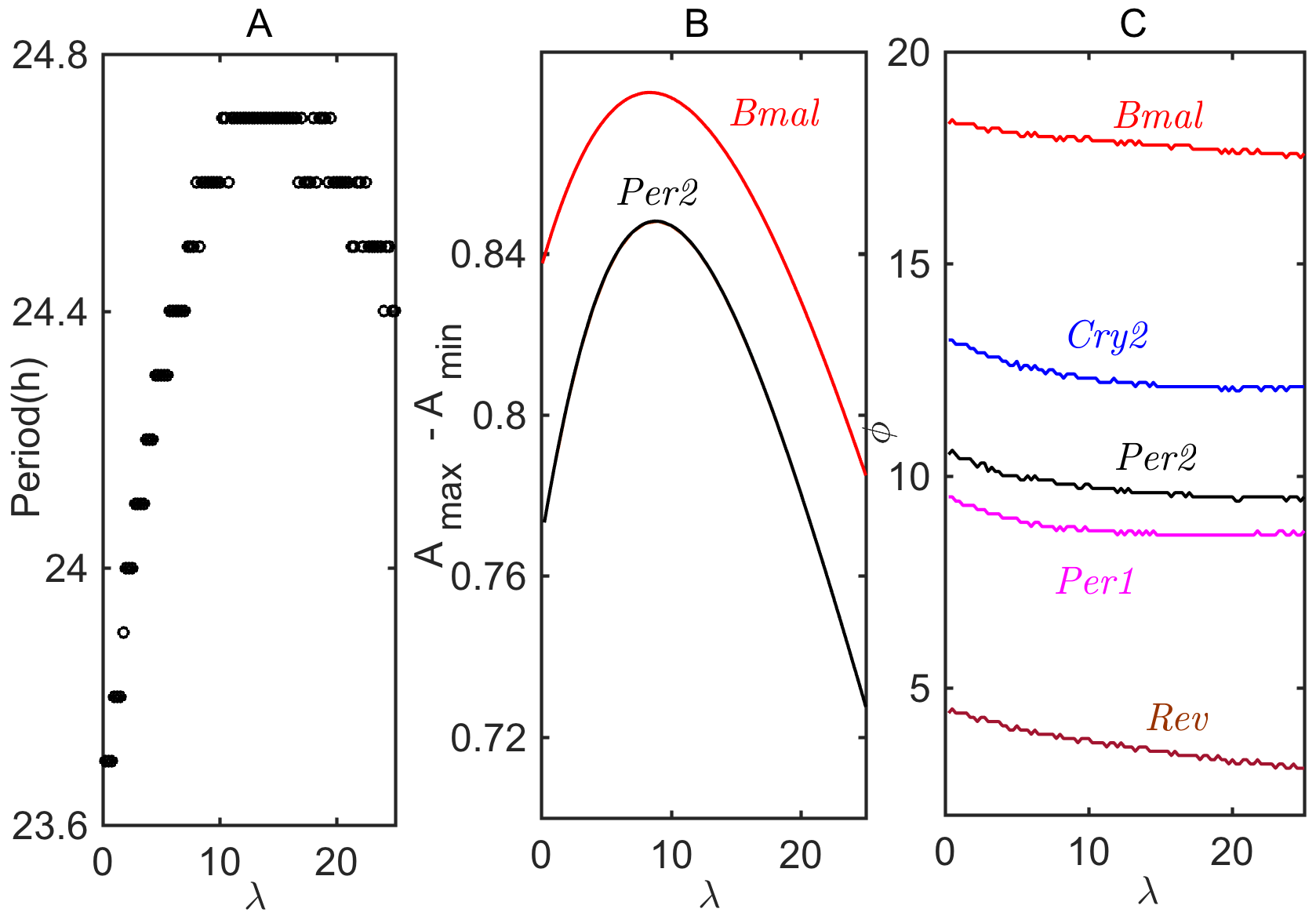
**

**Suppl. Figure S11.** Modulations of A) period, B) amplitude *A_max_−A_min_* (B), and C) phases *φ* of oscillations with increasing value of *λ*, the synthesis rate of *Per2AS*, in the mammalian circadian core-clock model by Mirsky *et al****.*** Compare this figure with Figure 4 of the main text. Note that because of the small amplitude of *Bmal,* we plotted 55*(*A_max_−A_min_*) in (B) for *Bmal.*
