# Supplementary material for: Modeling the interactions of sense and antisense Period transcripts in the mammalian circadian clock network
Source: PLoS Comput Biol. 2018 Feb 15;14(2):e1005957. doi: 10.1371/journal.pcbi.1005957 (PMC5831635; doi:10.1371/journal.pcbi.1005957)
Supplement: S12 Fig — (DOCX) [file pcbi.1005957.s018.docx]

**
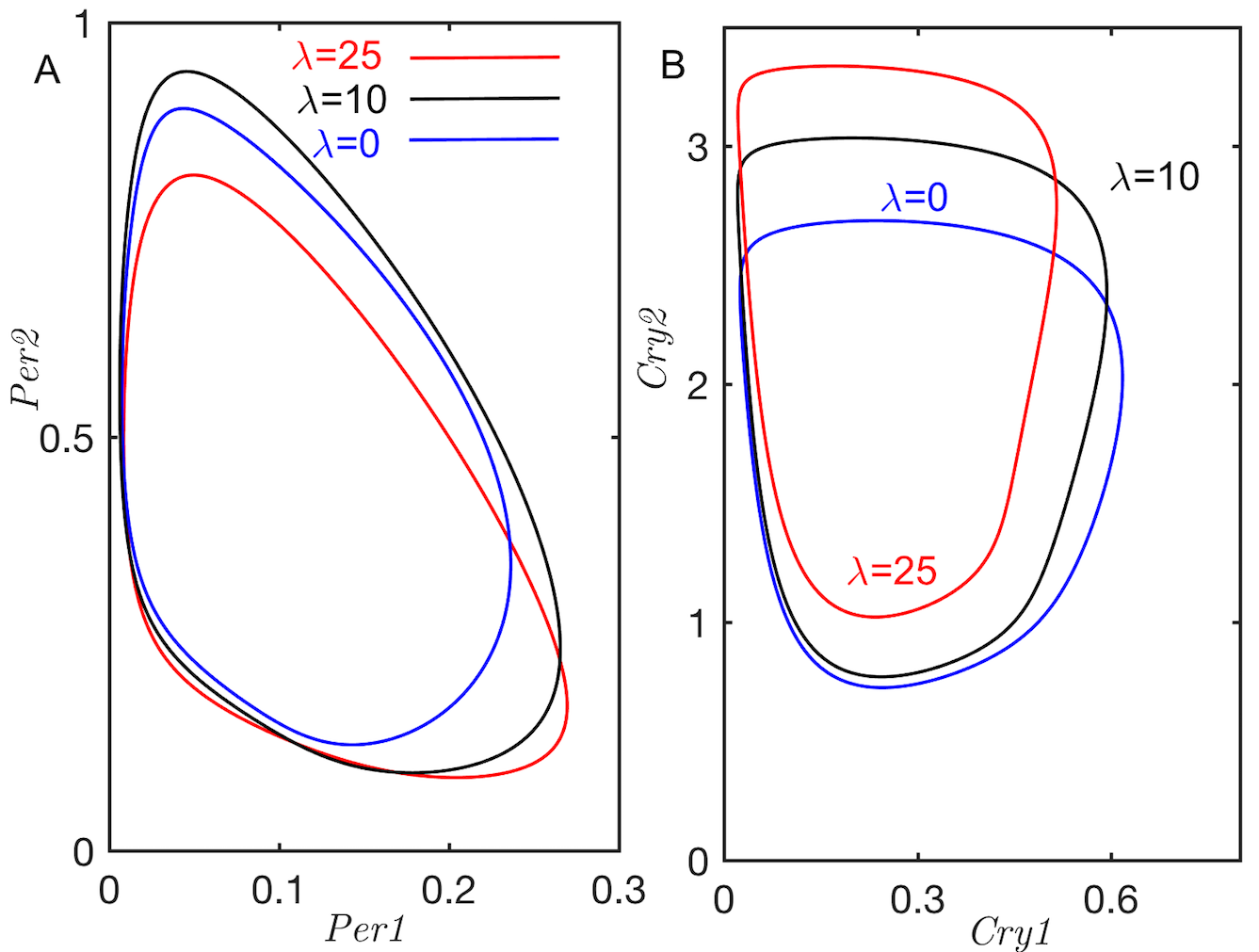
**

**Suppl. Figure S12.** (**A, B**) Circadian oscillations of the modified Mirsky *et al*. model, projected on the (*Per1,Per2*) and (*Cry1,Cry2*) phase-planes at different values of *λ*, the maximum rate of synthesis of antisense (*Per2AS*) RNA. Solid blue lines: oscillations in the absence of *Per2AS* ($\lambda=0)$. In panel A, when $\lambda=10$, the amplitudes of both *Per1* and *Per2* increase, although there is no direct effect of *Per2AS* on *Per1* expression. As the level of *Per2AS* is further increased$(\lambda=25)$, the amplitude of *Per2* drops, but the amplitude of *Per1* increases. In other words, panel A shows that the amplitudes of *PERIOD* transcripts, *Per1* and *Per2*, are independent and uncorrelated, when *Per2AS* is included in the model. Panel B also suggests that the amplitudes of *Cry1* and *Cry2* are anti-correlated as *Per2AS* levels increase.
