# Supplementary material for: Modeling the interactions of sense and antisense Period transcripts in the mammalian circadian clock network
Source: PLoS Comput Biol. 2018 Feb 15;14(2):e1005957. doi: 10.1371/journal.pcbi.1005957 (PMC5831635; doi:10.1371/journal.pcbi.1005957)
Supplement: S1 Table — (DOCX) [file pcbi.1005957.s019.docx]

**Suppl. Table S1. Model Parameter Values.**

| parameter | WT | Par_I | Par_II | Par_III | **Parameter description** |
| --- | --- | --- | --- | --- | --- |
| **Degradation rates for nuclear proteins or nuclear protein complexes [h^−1^]** | | | | | |
| *d_x1_* | 0.08 | 0.064 | 0.099 | 0.073 | CLOCK/BMAL |
| *d_x2_* | 0.06 | 0.057 | 0.055 | 0.048 | PER^*^_N_/CRY_N_ |
| *d_x3_* | 0.09 | 0.079 | 0.111 | 0.089 | PER_N_/CRY_N_ |
| *d_x5_* | 0.17 | 0.185 | 0.187 | 0.171 | REV-ERB_N_ |
| *d_x6_* | 0.12 | 0.076 | 0.104 | 0.109 | ROR_N_ |
| *d_x7_* | 0.15 | 0.11 | 0.176 | 0.12 | BMAL_N_ |
| **Degradation rates for mRNAs [h^−1^]** | | | | | |
| *d_y1_* | 0.3 | 0.358 | 0.338 | 0.29 | *Per* |
| *d_y2_* | 0.2 | 0.17 | 0.214 | 0.162 | *Cry* |
| *d_y3_* | 2 | 1.836 | 1.586 | 1.792 | *Rev-Erb* |
| *d_y4_* | 0.2 | 0.13 | 0.169 | 0.149 | *Ror* |
| *d_y5_* | 1.6 | 1.353 | 1.234 | 1.663 | *Bmal* |
| **Degradation rates for cytoplasmic proteins [h^−1^]** | | | | | |
| *d_z1_* | 0.23 | 0.208 | 0.22 | 0.236 | CRY_C_ |
| *d_z2_* | 0.25 | 0.194 | 0.293 | 0.22 | PER_C_ |
| *d_z3_* | 0.6 | 0.442 | 0.663 | 0.551 | PER_C_^*^ |
| *d_z4_* | 0.2 | 0.184 | 0.182 | 0.17 | PER_C_^*^/CRY_C_ |
| *d_z5_* | 0.2 | 0.207 | 0.254 | 0.151 | PER_C_/CRY_C_ |
| *d_z6_* | 0.31 | 0.274 | 0.35 | 0.274 | REV-ERB_C_ |
| *d_z7_* | 0.3 | 0.392 | 0.34 | 0.286 | ROR_C_ |
| *d_z8_* | 0.73 | 0.769 | 0.897 | 0.815 | BMAL_C_ |
| **Reaction rates for complex formation/dissociation** | | | | | |
| *kf_x1_* | 2.3 | 2.27 | 2.727 | 2.524 | CLOCK/BMAL formation **[h^−1^]**  CLOCK is assumed to be maintained at a constant (large) concentration in the nucleus. |
| *kd_x1_* | 0.01 | 0.009 | 0.011 | 0.01 | CLOCK/BMAL dissociation **[h^−1^]** |
| *kf_z4_* | 1 | 1.023 | 1.203 | 0.96 | PER_C_^*^/CRY_C_ formation **[(a.u.· h)^−1^]** |
| *kd_z4_* | 1 | 1.12 | 1.008 | 0.865 | PER_C_^*^/CRY_C_ dissociation **[h^−1^]** |
| *kf_z5_* | 1 | 1.162 | 1.054 | 0.919 | PER_C_/CRY_C_ formation **[(a.u.·h)^−1^]** |
| *kd_z5_* | 1 | 0.853 | 1.458 | 0.877 | PER_C_/CRY_C_ dissociation **[h^−1^]** |
| **Phosphorylation/dephosphorylation reaction rates [h^−1^]** | | | | | |
| *kph_z2_* | 2 | 1.768 | 2.132 | 2.199 | PER_C-_phosphorylation rate |
| *kd_phz3_* | 0.05 | 0.047 | 0.048 | 0.046 | PER_C_^*-^dephosphorylation rate |
| **Transcription rates [a.u.·h^−1^]** | | | | | |
| *V_1max_* | 1 | 0.943 | 0.978 | 0.913 | *Per* |
| *V_2max_* | 2.92 | 4.485 | 3.552 | 2.511 | *Cry* |
| *V_3max_* | 1.9 | 3.589 | 1.954 | 1.78 | *Rev-Erb* |
| *V_4max_* | 10.9 | 9.026 | 13.002 | 8.994 | *Ror* |
| *V_5max_* | 1 | 1.145 | 0.882 | 1.004 | *Bmal* |
| **Dissociation constants of transcription factors from gene loci [a.u.]** | | | | | |
| *k_t1_* | 3 | 3.639 | 3.594 | 2.403 | CLOCK/BMAL activation of *PER2* transcription |
| *k_i1_* | 0.9 | 0.825 | 0.801 | 0.921 | PER/CRY inhibition of *PER2* transcription |
| *k_t2_* | 2.4 | 1.733 | 2.893 | 2.146 | CLOCK/BMAL activation of *CRY* transcription |
| *k_i2_* | 0.7 | 0.538 | 0.68 | 0.684 | PER/CRY inhibition of *CRY* transcription |
| *ki_21_* | 5.2 | 6.979 | 4.494 | 3.801 | REV-ERB inhibition of *CRY* transcription |
| *k_t3_* | 2.07 | 1.818 | 2.246 | 1.796 | CLOCK/BMAL activation of *REV-ERB* transcription |
| *k_i3_* | 3.3 | 4.628 | 3.293 | 3.216 | PER/CRY inhibition of *REV-ERB* transcription |
| *k_t4_* | 0.9 | 0.851 | 0.987 | 1.015 | CLOCK/BMAL activation of *ROR* transcription |
| *k_i4_* | 0.4 | 0.548 | 0.447 | 0.393 | PER/CRY inhibition of *ROR* transcription |
| *k_t5_* | 8.35 | 9.256 | 7.775 | 6.677 | ROR activation of *BMAL* transcription |
| *k_i5_* | 1.94 | 1.908 | 1.487 | 1.893 | REV-ERB inhibition of *BMAL* transcription |
| **Transcription fold activation (dimensionless)** | | | | | |
| *a* | 12 | 17.508 | 10.877 | 14.77 | *Per* |
| *d* | 12 | 9.451 | 15.357 | 10.999 | *Cry* |
| *g* | 5 | 4.996 | 4.553 | 4.894 | *Rev-Erb* |
| *h* | 5 | 6.099 | 4.758 | 6.491 | *Ror* |
| *i* | 12 | 24.292 | 11.232 | 14.438 | *Bmal* |
| **Production rates of protein from mRNA [h^−1^]** | | | | | |
| *k_p1_* | 0.4 | 0.4 | 0.355 | 0.332 | PER_C_ |
| *k_p2_* | 0.26 | 0.287 | 0.288 | 0.3 | CRY_C_ |
| *k_p3_* | 0.37 | 0.568 | 0.327 | 0.4 | REV-ERB_C_ |
| *k_p4_* | 0.76 | 1.487 | 0.762 | 0.738 | ROR_C_ |
| *k_p5_* | 1.21 | 1.295 | 1.099 | 1.081 | BMAL_C_ |
| **Nuclear Import/Export rates [h^−1^]** | | | | | |
| *ki_z4_* | 0.2 | 0.385 | 0.226 | 0.192 | PER_C_^*^/CRY_C_ into nucleus |
| *ki_z5_* | 0.1 | 0.08 | 0.105 | 0.114 | PER_C_/CRY_C_ into nucleus |
| *ki_z6_* | 0.5 | 0.577 | 0.511 | 0.508 | REV-ERB_C_ into nucleus |
| *ki_z7_* | 0.1 | 0.106 | 0.092 | 0.101 | ROR_C_ into nucleus |
| *ki_z8_* | 0.1 | 0.098 | 0.1 | 0.102 | BMAL_C_ into nucleus |
| *ke_x2_* | 0.02 | 0.021 | 0.017 | 0.023 | PER^*^_N_/CRY_N_ into cytoplasm |
| *ke_x3_* | 0.02 | 0.026 | 0.016 | 0.024 | PER_N_/CRY_N_ into cytoplasm |
| **Hill exponents of transcription (dimensionless)** | | | | | |
| *b* | 5 | 4 | 6 | 8 | *Per*-activation by CLOCK/BMAL |
| *c* | 7 | 6 | 7 | 9 | *Per*-inhibition by PER/CRY |
| *e* | 6 | 1 | 14 | 7 | *Cry*-activation by CLOCK/BMAL |
| *f* | 4 | 2 | 8 | 4 | *Cry*-inhibition by PER/CRY |
| *f1* | 1 | 2 | 0 | 3 | *Cry*-inhibition by REV-ERB |
| *v* | 6 | 6 | 11 | 16 | *Rev-Erb*-activation by CLOCK/BMAL |
| *w* | 2 | 1 | 1 | 1 | *Rev-Erb*-inhibition by PER/CRY |
| *p* | 6 | 5 | 12 | 1 | *Ror*-activation by CLOCK/BMAL |
| *q* | 3 | 3 | 2 | 4 | *Ror*-inhibition by PER/CRY |
| *n* | 2 | 1 | 1 | 1 | *Bmal*-activation by ROR |
| *m* | 5 | 4 | 4 | 3 | *Bmal*-inhibition by REV-ERB |
| **Exogenous RNA levels [a.u.]** | | | | | |
| *y1_0_* | 0 | 0 | 0 | 0 | *Per* |
| *y2_0_* | 0 | 0 | 0 | 0 | *Cry* |
| *y3_0_* | 0 | 0 | 0 | 0 | *Rev-Erb* |
| *y4_0_* | 0 | 0 | 0 | 0 | *Ror* |
| *y5_0_* | 0 | 0 | 0 | 0 | *Bmal* |
| **Sense-antisense RNA interactions** | | | | | |
| *λ* | 1 | 1.005 | 0.774 | 0.862 | Maximum rate of synthesis of *Per2AS* **[a.u.** **h^−1^]** |
| *K_AS_* | 1 | 1.063 | 1.109 | 1.086 | *Per2AS* inhibition of *Per2* synthesis **[a.u.]** |
| *μ* | 1 | 1 | 1 | 1 | Magnitude of *Per2AS* effect on *Per2* synthesis (dimensionless) |
| *K_S_* | 0.1 | 0.101 | 0.1 | 0.077 | *Per2* inhibition of *Per2AS* synthesis **[a.u.]** |
| *d_AS_* | 2 | 1.669 | 2.126 | 1.409 | Degradation rate constant for *Per2AS* **[h^−1^]** |

*WT refers to the parameter values assigned in Relogio *et al.* [1], whereas ‘Par_I, _II, _III’ are sets of parameter values chosen by us, as described in Suppl. Text S4.

[1]. Relógio A, Westermark PO, Wallach T, Schellenberg K, Kramer A, Herzel H. Tuning the Mammalian Circadian Clock: Robust Synergy of Two Loops. *PLOS Comput. Biol.* **7**:e1002309 (2011).
